# Ambiguous structure determination from powder data: four different structural models of 4,11-di­fluoro­quinacridone with similar X-ray powder patterns, fit to the PDF, SSNMR and DFT-D

**DOI:** 10.1107/S2052252522004237

**Published:** 2022-05-14

**Authors:** Carina Schlesinger, Arnd Fitterer, Christian Buchsbaum, Stefan Habermehl, Michele R. Chierotti, Carlo Nervi, Martin U. Schmidt

**Affiliations:** a Institute of Inorganic and Analytical Chemistry, Johann Wolfgang Goethe University, Max-von-Laue-Straße 7, 60438 Frankfurt am Main, Germany; b Department of Chemistry and NIS centre, University of Torino, V. Giuria 7, Torino 10125, Italy

**Keywords:** ambiguous structures, structure determination from powder data, pair distribution function refinement, Rietveld refinement, solid-state NMR, lattice-energy minimization, DFT, quinacridone

## Abstract

Four different structural models, which all fit the same X-ray powder pattern, were obtained in the structure determination of 4,11-di­fluoro­quinacridone (C_20_H_10_N_2_O_2_F_2_) from unindexed X-ray powder data by a global fit. The models differ in their lattice parameters, space groups, *Z*, *Z*′, molecular packing and hydrogen bond patterns. The molecules form a criss-cross pattern in models A and B, a layer structure built from chains in model C and a criss-cross arrangement of dimers in model D. Nevertheless, all models give a good Rietveld fit to the experimental powder pattern with acceptable *R*-values. All molecular geometries are reliable, except for model D, which is slightly distorted. All structures are crystallochemically plausible, concerning density, hydrogen bonds, intermolecular distances *etc*. All models passed the *checkCIF* test without major problems; only in model A a missed symmetry was detected. All structures could have probably been published, although 3 of the 4 structures were wrong. The investigation, which of the four structures is actually the correct one, was challenging. Six methods were used: (1) Rietveld refinements, (2) fit of the crystal structures to the pair distribution function (PDF) including the refinement of lattice parameters and atomic coordinates, (3) evaluation of the colour, (4) lattice-energy minimizations with force fields, (5) lattice-energy minimizations by two dispersion-corrected density functional theory methods, and (6) multinuclear CPMAS solid-state NMR spectroscopy (^1^H, ^13^C, ^19^F) including the comparison of calculated and experimental chemical shifts. All in all, model B (perhaps with some disorder) can probably be considered to be the correct one. This work shows that a structure determination from limited-quality powder data may result in totally different structural models, which all may be correct or wrong, even if they are chemically sensible and give a good Rietveld refinement. Additionally, the work is an excellent example that the refinement of an organic crystal structure can be successfully performed by a fit to the PDF, and the combination of computed and experimental solid-state NMR chemical shifts can provide further information for the selection of the most reliable structure among several possibilities.

## Abstract 2

Four different structural models, which all fit the same X-ray powder pattern, were obtained in the structure determination of 4,11-di­fluoro­quinacridone (C_20_H_10_N_2_O_2_F_2_) from unindexed X-ray powder data by a global fit. All models are chemically sensible, but differ in their lattice parameters, space groups, *Z*, *Z*′, molecular packing and hydrogen bond patterns, and were investigated by fit to the pair-distribution function, solid-state NMR and lattice-energy minimizations.

## 1. Introduction

### 1.1. Ambiguous crystal structure solutions

Every crystal structure determination – from single-crystal or powder data – is an attempt to find an arrangement of atoms which fits the experimental diffraction pattern as well as possible, with the condition that the resulting structure is chemically sensible. Nevertheless, errors may always occur. In the early days of X-ray diffraction, sometimes the molecular geometry was considerably wrong, *e.g.* naphthalene assembled from cyclo­hexane rings (Bragg, 1921 ▸) or strongly bent or warped aromatic systems in quinacridone (Otaka, 1975 ▸), which is actually planar. Even single-crystal data can be of poor quality, so that the structure solution gives different possible structural models, which can all be refined more or less well. Nowadays, typical errors in single-crystal structure analyses include wrong space groups (see *e.g.* Baur & Kassner, 1992 ▸; Herbstein *et al.*, 2002 ▸; Marsh & Clemente, 2007 ▸; Henling & Marsh, 2014 ▸; Hempler *et al.*, 2017 ▸); overlooked, ignored or wrongly treated disorder; missing hydrogen atoms [*e.g.* Cp*CoCoCp* instead of Cp*CoH_3_CoCp* (Cp* = C_5_(CH_3_)_5_) (Kersten *et al.*, 1992 ▸)]; misplaced hydrogen atoms; or wrong atom assignments, *e.g.* N instead of C, Al instead of Si, or even [CuF_4_][ClF_6_] instead of [Cu(H_2_O)_4_][SiF_6_] (von Schnering & Vu, 1983 ▸). However, the overall crystal structure, *i.e.* the position and arrangement of ions and molecules, is generally close to being correct in single-crystal analyses.

**Figure 1:**
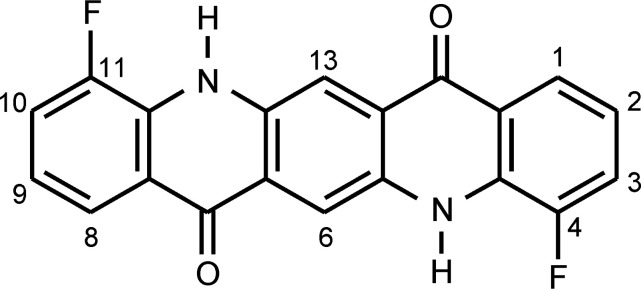
Structural sketch of 4,11-di­fluoro­quinacridone (DFQ), with numbering scheme. (In the parent compound quinacridone, both F atoms are replaced by H atoms.)

In X-ray powder diffraction, errors or uncertainties are more frequent, due to the limited information content of powder diffractograms. Generally, an X-ray powder pattern is considered to be characteristic for a certain crystal structure. Different molecular arrangements result in different patterns. Problems arise when the measured powder pattern is of limited quality, especially if the crystallite size is small and the powder diagram consists of only a few broad reflections. In such cases different structural models – even clearly wrong ones – can be in good agreement with the experimental pattern. One of the authors accidently observed such a case many years ago. On comparison of the structure data files of the β- and γ-phases of quinacridone (Fig. 1 ▸, H instead of F), it was observed that the powder pattern of the γ-phase could be easily fitted with the structure of the β-phase, although lattice parameters and molecular packing were considerably different: the β-phase exhibits a chain structure, the γ-phase a criss-cross structure. Even the Rietveld refinement seemed successful and the resulting structure was sensible in every aspect including the hydrogen-bonding pattern. Nevertheless, the structure was completely wrong, with incorrect lattice parameters, space group, and molecular packing and conformation (Buchsbaum & Schmidt, 2007 ▸). This should serve as a warning that a good fit to the powder pattern is not a proof that the structure is actually the correct one.

The usual approach for structure determination from powder data starts with the indexing of the powder pattern, *i.e.* the determination of the lattice parameters from the reflection positions. If the indexing is successful, and the lattice parameters are correct, then usually only one (or a few quite similar) possible crystal structure exists which (1) gives a good fit to the powder diffractogram and (2) is chemically sensible, *i.e.* the molecular geometry is reliable, the hydrogen bond system is sensible, the residual electron density is not too high, and the structure does not show voids or unrealistically close contacts.

The number of possible crystal structures fulfilling these two criteria increases considerably if the structure is solved without prior indexing, *i.e.* if the lattice parameters and space group are not known beforehand. Structure solution from powder data without prior knowledge of lattice parameters and space group is very challenging. All classical structure solution methods, such as real-space methods, direct methods, charge flipping and Patterson methods require the lattice parameters as input, *i.e.* they do not work without a successful indexing. Very few organic structures have been solved from scratch by crystal structure prediction with a global optimization of the lattice energy using force-field methods (*e.g.* Schmidt & Dinnebier, 1999 ▸; Schmidt *et al.*, 2005 ▸; Hammer *et al.*, 2011 ▸) or quantum mechanical methods (Day *et al.*, 2006 ▸; Zhu *et al.*, 2016 ▸; Askin *et al.*, 2019 ▸; Bhardwaj *et al.*, 2019 ▸) or using statistical potentials (Hofmann & Kuleshova, 2005 ▸; Schmidt *et al.*, 2007 ▸). According to our experience, most predicted structures which have favourable lattice energies exhibit very different simulated powder patterns, and there is usually only one structure which simultaneously has a good energy and fits the powder data well.

The situation changes if the structure is solved from powder diffraction data alone, without taking the lattice energy into account. In this case the number of different possible structures is expected to be much higher, and the probability to find multiple solutions, which fit the powder data similarly well, will increase, especially if the powder data are of limited quality. However, it is very challenging or difficult to solve a crystal structure using only the molecular geometry and the powder diffractogram as input, if the powder data cannot be indexed. In this case, a global fit to the powder data must be performed, for example, starting from a large number of random structures with different lattice parameters and space groups, which are then fitted to the powder data. There have been some attempts to develop such a method (*e.g.* Padgett *et al.*, 2007 ▸; Rapallo, 2009 ▸), but to our knowledge none of these methods have become widely used. Apart from the high calculation effort, one obstacle is the difficulty of comparing simulated and experimental powder patterns: as long as the lattice parameters deviate, the calculated reflections positions do not match the experimental ones, and the usual fitting procedure by minimization of the difference curve between calculated and experimental powder data does not work.

**Figure 2:**
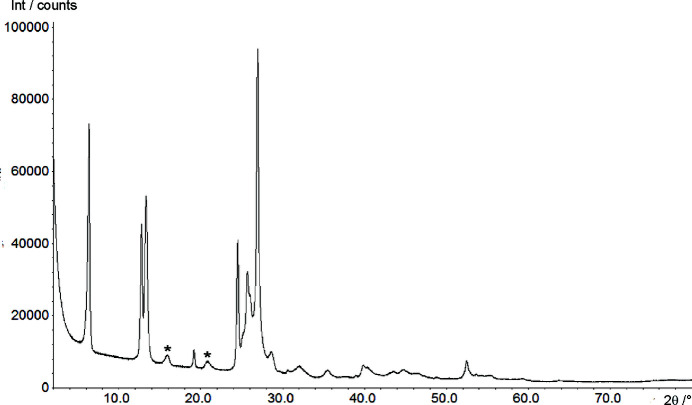
X-ray powder pattern of DFQ, measured with Cu *K*α_1_ radiation. The stars denote two single-indexed broad reflections. For discussion see Section 3.3.

Habermehl *et al.* (2014 ▸, 2022 ▸) have developed a method that uses the generalized similarity measure *S*_12_ based on cross-correlation functions (de Gelder *et al.*, 2001 ▸) to compare simulated and experimental powder patterns. The method is called *FIDEL-GO* (fit with deviating lattice parameters, global optimization) and provides a very elaborate, automated structure solution and refinement process, starting from a huge number of random structures with random lattice parameters in various space groups. We applied this method to solve crystal structures of organic compounds from non-indexable powder data. Already in one of the first application examples, we obtained multiple structure solutions. The compound 4,11-di­fluoro­quinacridone (DFQ) is shown in Fig. 1 ▸. The compound is has low crystallinity, and the powder pattern consists of only a few sharp peaks and some humps (Fig. 2 ▸); consequently, the pattern could not be indexed in a reliable way. Hence, the structure was solved by a *FIDEL-GO* fit. The structure solution and refinement process resulted in four different structural models, which fitted the powder pattern similarly well. Rietveld refinements were successful on all four models in terms of acceptable *R*-values and a smooth difference curve of calculated and experimental powder patterns. The structures are totally different, with different lattice parameters, space groups, *Z*, *Z*′ and a different molecular packing: two criss-cross structures, one chain structure and one criss-cross packing of dimers. All structures were crystallochemically plausible.

In order to determine which one of the structures is correct, six different methods were applied: (1) careful user-controlled Rietveld refinement under identical conditions for all four structural models; (2) structure refinement by a fit to the pair distribution function (which is a new, valuable approach for organic compounds; an introduction is given in Section 1.3); (3) evaluation of the colour; (4) lattice-energy minimization with force-field methods; (5) lattice-energy minimization with quantum-mechanical methods [dispersion-corrected density functional theory (DFT-D)]; (6) solid-state NMR (SSNMR) experiments and comparison of the chemical shifts with values calculated by the GIPAW method after DFT-D geometry optimization.

In this paper, we report on these six investigations. Additionally, we try to explain why four very different structural models could result in similar X-ray powder patterns. Finally, we discuss which structure should be regarded as the correct one.

### 1.2. On 4,11-di­fluoro­quinacridone

DFQ is a non-commercial derivative of quinacridone (see Fig. 1 ▸). Quinacridone and many of its derivatives are commercially used as organic pigments for the coloration of lacquers and coatings, plastics, and printing inks (Hunger & Schmidt, 2018 ▸). They offer very good light and weather stability, as well as resistance to solvent and migration. The compounds are fully insoluble in water and all solvents. Most quinacridone pigments have shades from red to violet. In contrast, DFQ is orange. Crystal structures of several industrial quinacridone pigments have been determined by single-crystal analyses (Paulus *et al.*, 1989 ▸; Mizuguchi *et al.*, 2002*a* ▸,*b* ▸; Senju *et al.*, 2005*a* ▸,*b* ▸, 2006 ▸; Nishimura *et al.*, 2006 ▸; Paulus *et al.*, 2007 ▸). Some quinacridone structures were determined by X-ray powder diffraction (Schlesinger *et al.*, 2020 ▸; Habermehl *et al.*, 2022 ▸), crystal structure prediction with subsequent Rietveld refinement (Leusen, 1996 ▸; Paulus *et al.*, 2007 ▸) or even electron diffraction (Gorelik *et al.*, 2016 ▸). The crystal structure of DFQ was not known, hitherto. Its powder pattern is shown in Fig. 2 ▸.

### 1.3. Structure refinement by fit to the pair distribution function

The pair distribution function (PDF) gives the probability *G*(*r*) of finding a pair of atoms with an interatomic distance *r*, summed over all atom–atom distances, and weighted by their scattering power. The PDF can be derived from carefully measured powder diffraction data, using the total scattering information, *i.e.* not only the Bragg peaks, but also the diffuse scattering. The strength of PDF analysis is the identification of the local structure instead of providing the average crystal structure obtained from the Bragg peaks (Billinge, 2019 ▸). From a given structural model the PDF can be calculated according to Egami & Billinge (2012 ▸):

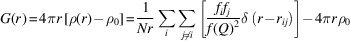

with the scattering power *f_i_* and *f_j_* of the atoms *i* and *j*, respectively, the Dirac delta function δ, the number of atoms *N*, the atom-pair density ρ(*r*) as a function of the distance *r*, and the average atom-pair density ρ_0_.

By comparison of the simulated PDF curve of a structural model to experimental data, a refinement of structural data by a PDF fit is possible. For such a refinement, the PDF curve should have a good resolution in *r*, which requires powder data with a high *Q*_max_, *i.e.* a measurement with a short wavelength (*e.g.* 0.1–0.4 Å) and a large 2θ range, *i.e.* with synchrotron radiation. (In contrast, for a qualitative identification of phases and their local structures via their PDFs, laboratory data are frequently sufficient.)

In organic compounds, intramolecular atom–atom distances result in sharp PDF signals, whereas intermolecular atom–atom distances (soft interactions with large vibrational amplitudes) cause broad signals. Hence, a reliable simulation of the PDF curves should differentiate between intramolecular and intermolecular atom–atom distances (Rademacher *et al.*, 2012 ▸). This is carried out using two different isotropic displacement parameters [‘Prill method’ (Prill *et al.*, 2015 ▸)]. With this approach, organic crystal structures can be successfully refined by a fit to the PDF (Prill *et al.*, 2016 ▸). Even a structure solution from scratch is possible, using a global fit to the PDF data (Habermehl *et al.*, 2021*a* ▸,*b* ▸, 2022 ▸; Schlesinger *et al.*, 2021 ▸).

### 1.4. Combination of different methods for structure solution, refinement and verification

If a difficult crystal structure cannot reliably be determined by a single analytical method, *e.g.* single-crystal X-ray diffraction, X-ray power diffraction or electron diffraction, then it is advantegeous to combine different methods in order to obtain as much experimental information as necessary.

It has been demonstrated that the structure determination process is significantly enhanced in terms of speed and reliability by combining powder X-ray diffraction (PXRD) data with solid-state NMR (SSNMR) and DFT information (Widdifield *et al.*, 2020 ▸). This combined approach has been extensively used in solving the structures of polymorphic (Meejoo *et al.*, 2003 ▸) and tautomeric (Schmidt *et al.*, 2011 ▸; Gumbert *et al.*, 2016 ▸; Smalley *et al.*, 2022 ▸) systems as well as of salts, cocrystals and solvates (Dudenko *et al.*, 2013 ▸, 2020 ▸; Braga *et al.*, 2013 ▸; Corlett *et al.*, 2019 ▸). The usefulness and efficacy of this approach has been demonstrated in many fields of science including, for example, pharmaceuticals (Smith *et al.*, 2001 ▸; Tatton *et al.*, 2018 ▸; Rahal *et al.*, 2021 ▸), biology (Reddy *et al.*, 2015 ▸; Hughes *et al.*, 2017 ▸) and pigments (Tapmeyer *et al.*, 2020 ▸). In particular, SSNMR, while also useful for improving the quality of structures solved by single-crystal X-ray diffraction (Rossi *et al.*, 2018 ▸; Bernasconi *et al.*, 2020 ▸), is able to provide the structure solution, even if SSNMR is combined only with DFT or crystal structure prediction calculations (Elena & Emsley, 2005 ▸; Salager *et al.*, 2010 ▸; Thureau *et al.*, 2019 ▸; Bravetti *et al.*, 2022 ▸). Instead of DFT-D, statistical potentials can be used (Hofmann, 1998 ▸; Hofmann & Apostolakis, 2003 ▸; Hofmann *et al.*, 2004 ▸).

In this paper we go a slightly different way. At first, we tried to get as much information as possible from the X-ray powder data. DFQ was used as a test example in the method development of the *FIDEL-GO* procedure to solve crystal structures from unindexed powder data without using any other experimental information. When it became apparent that, for the case of DFQ, this method reaches its limits, and the structure solution remained ambiguous, we applied additional analytical methods: Rietveld refinement, refinement to the PDF, evaluation of the colour, lattice-energy minimizations with force-field and DFT-D methods, and SSNMR experiments coupled with the calculations of NMR shifts.

## 2. Experimental

### 2.1. Materials

DFQ was obtained from Clariant (now Heubach; Frankfurt–Höchst, Germany).

### 2.2. X-ray powder diffraction

#### 2.2.1. Powder diffraction for structure solution and Rietveld refinement

The DFQ sample was prepared between two polymer films. PXRD data were recorded at room temperature with Cu *K*α_1_ radiation in transmission mode on a Stoe Stadi-P diffractometer equipped with a primary Ge(111) monochromator and a linear position-sensitive detector. The sample was rotated during the measurement. Data were collected in a 2θ range from 2.00 to 79.99° with a resolution of 0.01° per step, resulting in 7800 data points. The software *WinX^Pow^* (Stoe & Cie, 2005 ▸) was used for data collection and reduction.

#### 2.2.2. Powder diffraction for pair distribution function refinement

For the measurement of the PDF data, the sample was placed in glass capillaries with a 1 mm diameter and sealed with clay. Synchrotron measurements were carried out at the Diamond Light Source in Didcot (UK) at the beamline I15-1 using a Perkin Elmer detector. The capillaries were rotated in a 10 Hz capillary spinner. A monochromatic incident X-ray beam was used with a standard size at the sample of 700 µm × 150 µm, conditioned using a bent Laue monochromator to provide an energy of 76 keV (λ = 0.1631 Å). Two measurements were made, one at room temperature the other at 173 K. For both measurements, the background was measured using an empty capillary. The collected 2D synchrotron powder diffraction data were automatically converted to a 1D dataset using *DAWN* (Filik *et al.*, 2017 ▸).

#### 2.2.3. Obtaining the pair distribution function

The PDF G(*r*) was obtained from the synchrotron powder data by background correction, normalization and Fourier transformation using the program *PDFgetX3* (Juhás *et al.*, 2013 ▸). To exclude artefacts and insufficient statistics in the high 2θ range, the data were truncated at a finite maximum value of the momentum transfer *Q*_max_ = 15.02 Å^−1^ for the room-temperature data. For the low-temperature data, it was necessary to diminish the cut-off value to *Q*_max_ = 13.51 Å^−1^.

### 2.3. Structure solution by global optimization with *FIDEL-GO*

The molecular geometry of DFQ was obtained by optimization on the HF/6-31G** level using *Gaussian09* (Frisch *et al.*, 2009 ▸). In the structure solution, the molecule was treated as rigid. With *FIDEL-GO*, random starting structures in various space groups were generated. The space groups were selected based on space group frequency. The DFQ molecule has 2/*m* symmetry. According to Pidcock *et al.* (2003 ▸), molecules with 2/*m* symmetry are located on crystallographic inversion centres in 95% of the crystal structures. Therefore, the investigated space groups were *P* 1 (*Z* = 1), *P*2_1_/*c* (*Z* = 2), *C*2/*c* (*Z* = 4) and *Pbca* (*Z* = 4), each with a molecule on the inversion centre. Additional runs were performed with molecules on the general position in *P*1 (*Z* = 1), *P*2_1_ (*Z* = 2), *P*2_1_/*c* (*Z* = 4) and *P*2_1_2_1_2_1_ (*Z* = 4); these runs also covered other rare crystal symmetries such as *C*2/*c* (*Z* = 4) or *Pbcn* (*Z* = 4), both with molecules on the twofold axes. Furthermore, calculations were run in *C*2/*m* (*Z* = 2) with molecules on 2/*m* positions. Random starting values were used for the lattice parameters, the molecular orientation and the molecular position (if not constrained by symmetry). After removing the structures with overlapping molecules, about 21 million structures remained. These structures were fitted to the experimental powder data using the similarity measure S_12_ in an elaborated automated multi-step process, which includes ranking according to the similarity to the powder pattern, clustering and removal of duplicates. A detailed description of the *FIDEL-GO* procedure is given by Habermehl *et al.* (2022 ▸). The most promising structure candidates were subjected to an automated Rietveld refinement using *TOPAS Academic* (version 4.2; Coelho, 2018 ▸) controlled by the program *FIDEL-GO*. The Rietveld refinements included the lattice parameters, background, peak profile, peak width anisotropy and all atomic coordinates, using restraints on all bond lengths, bond angles and the molecular planarity.

### 2.4. Rietveld refinement

The four structural models from the global fit with *FIDEL-GO* were again refined by the Rietveld method in a user-controlled procedure using *TOPAS Academic*. In order to ensure the comparability of the refinements of the different models, a strictly identical refinement procedure was applied for all models A–D. At first, a Pawley fit (Pawley, 1981 ▸) was carried out. The peak profile was described by the full axial divergence model using the method of Cheary & Coelho (1998 ▸). The background defined by 20 Chebyshev polynomial terms, scale factor, zero-point and lattice parameters were also refined. Crystallite size and strain broadening by means of Gaussian and Lorentzian component convolution were included and refined. Since the pattern contained a mixture of sharp and broad reflections, the peak width anisotropy was described by spherical harmonics of the sixth order. Subsequently, the atomic positions and one isotropic displacement parameter were refined. For the molecular geometry, restraints were applied to all bond lengths, bond angles and for the molecular planarity. The restraint values were derived from the molecular geometry optimized by DFT-D. Preferred orientation was neither observed nor refined.

### 2.5. Structure refinement by fit to the PDF

The four Rietveld-refined structural models were refined by a fit to the PDF curve G(*r*). For all four models, an identical molecular geometry was used, which was obtained by force-field optimization of model A (for details on the force field, see next section).

The refinement to the PDF was carried out with *TOPAS Academic* (version 6; Coelho, 2018 ▸). The molecular geometry was described as rigid body using the *z*-matrix formalism, including the hydrogen atoms. A PDF refinement can be performed with different sections (*r*-ranges) of the PDF curve. For DFQ, an *r*-range of 1.2–35.0 Å was found to be optimal. Since a simultaneous PDF refinement of all parameters bears the risk of divergence, refinement of the four models was performed in a 14-step sequence. Each step comprised a different combination of parameters, which were simultaneously refined. In the first steps the scale factor, dampening effects of the sample described by Gauss-functions, zero-point, and one intermolecular and one intramolecular isotropic displacement parameter were refined, while keeping the molecular position and orientation fixed. Subsequently, the lattice parameters were refined while the already refined parameters of the first steps were kept fixed. In the next steps the molecular position and orientation were refined, whereas the lattice parameters were fixed. These steps are iteratively repeated until the calculated PDF curve is in good agreement with the experimental one. This procedure ensures a robust refinement. In the last refinement step all variables are refined simultaneously. To ensure the comparability of the results, all models were refined with an identical procedure.

A second set of PDF refinements was performed in a similar way, but the molecular geometry was described using restraints instead of a rigid body given by a *z*-matrix. Here, all atomic coordinates, including hydrogen atoms, were refined.

Two sets of PDF refinements were performed, one using the room-temperature measurement, the second using the low-temperature (173 K) data.

### 2.6. Lattice-energy minimizations using force fields

Lattice-energy minimizations were performed with *Materials Studio* (version 4.4; Dassault Systèmes, 2008 ▸), using the Dreiding force field with the van der Waals interactions given by a 6–12 potential (Mayo *et al.*, 1990 ▸). Atomic charges were calculated using the method by Gasteiger & Marsili (1980 ▸). At first, only the molecular geometry was optimized, in the later steps additionally the lattice parameters. The space group symmetry was maintained throughout.

### 2.7. Lattice-energy minimizations by DFT-D

The four structural models were optimized by lattice-energy minimizations with DFT-D. Two series of DFT-D calculations were performed, one with *GRACE* (Neumann *et al.*, 2008 ▸), the other with *Quantum Espresso* (Giannozzi *et al.*, 2009 ▸, 2017 ▸).

*GRACE* uses the *VASP* code (Kresse & Hafner, 1993 ▸, 1994 ▸; Kresse & Furthmüller, 1996*a* ▸,*b* ▸) for the DFT calculations, and adds a dispersion correction after each DFT step. The Perdew–Burke–Ernzerhof functional (PBE, Perdew *et al.*, 1996 ▸) was used. The dispersion correction was performed according to Neumann & Perrin (2005 ▸).

The second series was run using the *Quantum Espresso* package (version 6.4.1), using the non-local vdW-DF2 (Lee *et al.*, 2010 ▸) with PBE pseudopotentials from *PS Library* (version 1.0.0; Dal Corso, 2014 ▸), imposing an energy cut-off of 60 Ry.

All calculations were run twice: in the first one, the lattice parameters were taken from the Rietveld refinement and kept fixed, optimizing only the atomic positions. In the second one, the lattice parameters were optimized too.

### 2.8. Solid-state NMR investigations

#### 2.8.1. Calculation of SSNMR spectra

On the basis of the structures optimized by *Quantum Espresso* with fixed lattice parameters, the NMR chemical shifts were calculated with the Gauge-Including Projected Augmented-Wave (GIPAW) (Yates *et al.*, 2007 ▸), with an energy cutoff of 80 Ry, in a similar way to the previously published procedure (Franco *et al.*, 2013 ▸). The theoretical chemical shifts (δ) were calculated from the corresponding absolute magnetic shielding (σ) values by δ_calc_ = σ_ref_ − σ, where σ_ref_ is the shielding of the reference substance DABCO, calculated at the same level. σ_ref_ was obtained by plotting the experimental chemical shifts, δ_exp_ against σ. A linear regression model with fixed slope of −1 resulted in σ_ref_ values of 120.59 for ^13^C.

#### 2.8.2. SSNMR measurements

The ^13^C CPMAS spectrum of DFQ was acquired with a Jeol ECZR 600 instrument, operating at 600.17 and 150.91 MHz, for ^1^H and ^13^C nuclei, respectively. The powder was collected from the batch and used without further preparation to fill the rotor (3.2 mm outer diameter; 60 µl volume). The ^13^C CPMAS spectrum was acquired at a spinning speed of 20 kHz, using a ramp cross-polarization pulse sequence with an ^1^H 90° pulse of 2.19 µs, and a contact time of 3.5 ms. An optimized recycle delay of 8.1 s was used for 700 scans. A two-pulse phase modulation (TPPM) decoupling scheme was used, with a radiofrequency field of 108.5 kHz. ^1^H and ^19^F MAS, and ^19^F–^13^C CPMAS spectra of DFQ were acquired with Bruker Avance II 400 Ultra Shield instrument, operating at 400.23, 376.59 and 100.63 MHz for ^1^H, ^19^F and ^13^C nuclei, respectively. ^1^H and ^19^F MAS were performed in a 2.5 mm rotor (10 µl volume) at a spinning speed of 32 kHz with the *DEPTH* sequence (π/2–π–π; ^1^H 90° = 2.5 µs; ^19^F 90° = 3.2 µs; 16 scans; optimized recycle delays: ^1^H 5.3 s and ^19^F 10 s) for the suppression of the probe background signal. The ^19^F–^13^C CPMAS spectrum was acquired in a 4 mm outer diameter (80 µl volume) rotor spun at 12 kHz with an ^19^F–^13^C ramp cross-polarization pulse sequence (^19^F 90° = 4.0 µs; contact time = 3 ms; optimized recycle delay = 10 s; number of scans = 6200). A TPPM decoupling scheme was used, with a radiofrequency field of 62.5 kHz. ^1^H, ^19^F and ^13^C chemical shift scales were calibrated through adamantane (^1^H signal at 1.87 p.p.m.), polytetra­fluoro­ethyl­ene (^19^F signal at −122 p.p.m.) and glycine (^13^C methyl­enic signal at 43.7 p.p.m.) used as external standards.

## 3. Results and discussion

### 3.1. Structure solution by a global fit to the powder data

The X-ray powder pattern of DFQ exhibits only about 9 sharp reflections and about 15 broad peaks or humps (see Fig. 2 ▸). The low quality of the diagram is not caused by the diffractometer: the sample was measured in transmission geometry on a diffractometer which provides a good 2θ resolution; and the measurement was performed with a sufficient amount of sample in the X-ray beam and a long counting time resulting in a diagram with good counting statistics. The low number of visible reflections and the broad width of several reflections is caused by the sample itself, which is of limited crystallinity. Reliable indexing was not possible.

Therefore, the structure was solved from scratch by a global fit to the powder data using the program *FIDEL-GO* (Habermehl *et al.*, 2022 ▸). The fit started from about 21 million random structures in different space groups. After several steps of ranking, fitting, clustering and evaluation, 122 structure candidates remained. A total of 18 of these structures were subjected to a fully automated Rietveld refinement with *TOPAS*, controlled by *FIDEL-GO*. Five structures were evaluated by DFT-D geometry optimization. Four structures remained, which were denominated A, B, C and D (see Table 1 ▸). Their Rietveld plots from the automated refinements are given in Fig. S1 of the supporting information.

### 3.2. The four structural models

All four structural models A–D were chemically sensible. This is astonishing because the structures were solved and refined only by a fit to the powder pattern, without consideration of hydrogen bonds, without anti-bumb restraints and with no regard for any other intermolecular interaction. Nevertheless, in all structures, the molecules form a dense packing with sensible intermolecular distances and with no unreasonably short intermolecular contacts. Even all hydrogen bond patterns are reliable; all molecules are connected by four N—H⋯O=C hydrogen bonds (two donors, two acceptors). The calculated density of A–D deviates by less than 5%, and the molecular volume *V*/*Z* matches exactly the value calculated by Hofmann’s volume increments (*V*/*Z* = 358.5–361.4 Å^3^ for A–D, 361.2 Å^3^ from Hofmann’s values, corrected by the empirical factor of 0.91 for organic pigments). From the viewpoint of a crystallographer or chemist, all four structures look plausible. And, of course, all structures give a good fit to the powder data.

**Figure 3:**
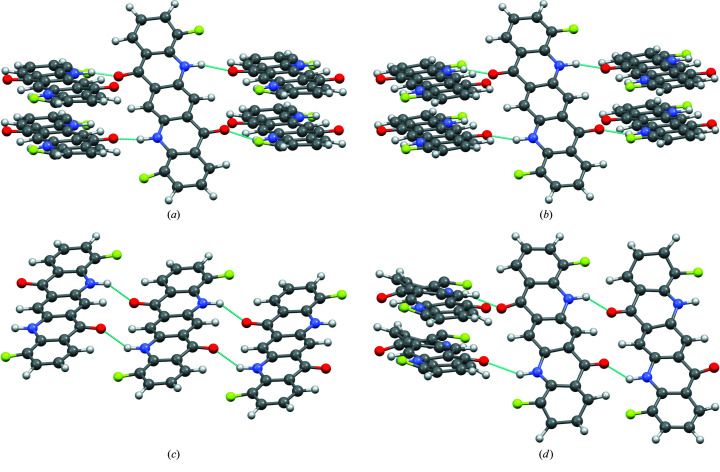
Molecular geometry and hydrogen bond patterns in the models (*a*) A, (*b*) B, (*c*) C and (*d*) D, after user-controlled Rietveld refinement. Colour code in all drawings: C grey, H white, F yellow, N blue, O red. Hydrogen bonds are drawn as blue dotted lines.

Nevertheless, the structures differ in their space groups, *Z*, *Z*′, lattice parameters, molecular packing and hydrogen-bond topology (see Fig. 3 ▸).

Model A: *P*2_1_/*c* (*Z* = 4, *Z*′ = 1, molecule on the general position). Each molecule is connected to four neighbouring molecules by one hydrogen bond. The molecules form a criss-cross pattern.

Model B: *P*2_1_/*c* (*Z* = 2, *Z*′ = 0.5, molecule on 1). As in model A, each molecule is connected to four other molecules in a criss-cross pattern. Model B differs from model A by a unit cell of half the size, and their molecular site symmetry. In model B, the molecules are situated on an inversion centre, whereas in model A they are shifted sidewards by 0.22 Å along the long axis of the molecules which results in a loss of the corresponding crystallographic inversion centres and a doubling of the lattice parameter. Such a criss-cross arrangement with molecules on inversion centres is known for the γ-phase of unsubstituted quinacridone (Paulus *et al.*, 1989 ▸, 2007 ▸; Potts *et al.*, 1994 ▸; Mizuguchi *et al.*, 2002*a* ▸).

Model C: *P* 1 (*Z* = 1, *Z*′ = 0.5, molecule on 1). The molecule is connected to only two neighbouring molecules by a double hydrogen bond each, resulting in a chain. Within the chains, the molecules form small steps. All chains are parallel. This structure type is known for the α^I^-phase of unsubstituted quinacridone (Paulus *et al.*, 2007 ▸), 2-methyl­quinacridone (Schlesinger *et al.*, 2020 ▸) and 2,9-di­methyl­quinacridone (Paulus *et al.*, 1989 ▸; Mizuguchi *et al.*, 2002*b* ▸).

Model D: *P*2_1_/*c* (*Z* = 4, *Z*′ = 1, molecule on the general position). Model D is a ‘mixture’ of model A (or B) with model C. Each molecule is connected to three neighbouring molecules: on one side to a single neighbour by two hydrogen bonds, on the other side to two neighbours by a single hydrogen bond [see Fig. 3 ▸(*d*)]. This topology can be described as a criss-cross pattern of dimers. Although this structure is crystallochemically plausible, it has not been observed for any quinacridone yet, and, to our knowledge, for no other organic pigment.

### 3.3. Rietveld refinements

Prior to the Rietveld refinements, we performed Pawley fits. The four structural models A–D have different space groups and lattice parameters. Hence, they might show differences already in a Pawley fit. However, all models gave excellent Pawley fits (see Fig. S2). Hence, none of the models could be ruled out by a Pawley fit.

**Figure 4:**
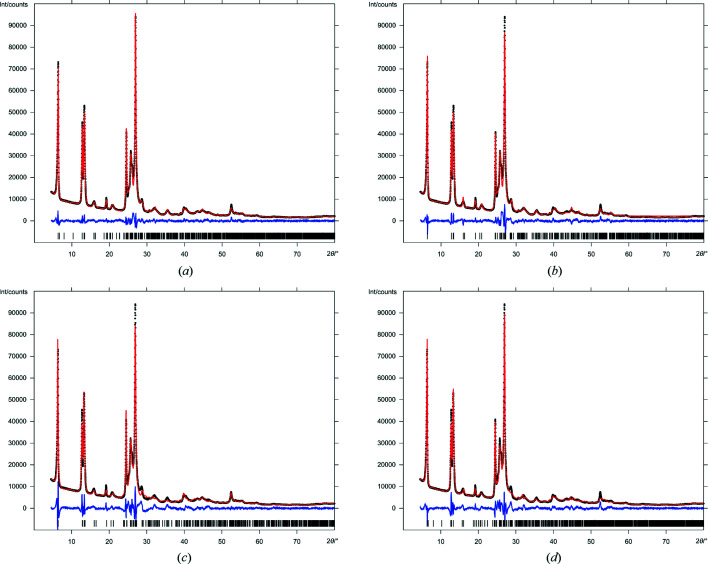
Rietveld plots of structural models A–D. Observed intensities are denoted as black dots, calculated intensities as a red line, difference curve below in blue, vertical ticks represent the reflection positions.

The four structural models A–D were subjected to user-controlled Rietveld refinements. All structures gave a fairly good fit to the powder data with a quite smooth difference curve and acceptable *R*-values (Table 2 ▸, Fig. 4 ▸). Some of the remaining differences between simulated and experimental curves result from difficulties in fitting the strongly anisotropic peak widths, as is visible in the difference curve which has positive spikes directly beside negative spikes.

Models C and D result in a slightly worse fit than A and B. The molecules of C and D become slightly distorted. The distortion during the refinement can be quantified by the root mean-square Cartesian deviation (RMSCD) of all non-hydrogen atoms (van de Streek & Neumann, 2010 ▸). The RMSCD of model D is 0.208 Å, whereas the value of model B is as low as 0.057 Å. However, such small distortions are common when using powder data of such limited quality; hence, the distortion is not an argument against models C or D.

**Figure 5:**
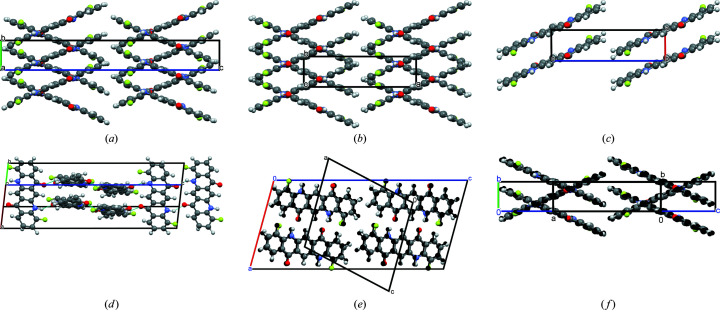
Crystal structures of models (*a*) A, (*b*) B, (*c*) C and (*d*) D, after user-controlled Rietveld refinements. (*e*) Overlay of the models A (coloured) and B (black), view direction [010]. (*f*) Overlay of A (coloured) and B (black), view direction [100] for A and [001] for B.

During the Rietveld refinement, models A and B became very similar [Figs. 5 ▸(*e*) and 5(*f*)]. Model A is described in the space group *P*2_1_/*c*, *Z* = 4, but its structure is very close to having the higher symmetry space group *P*2_1_/*c*, *Z* = 2, *Z*′ = 0, 5. The resulting lattice parameters are similar to those of B. Nevertheless, model A gives a better fit to the powder data than model B, with a slightly smoother difference curve, and significantly lower *R*-values (see Table 2 ▸). On the other hand, model A has double the number of atoms per asymmetric unit, hence double the number of parameters for the atomic positions, which automatically leads to an improved fit. Consequently, from the Rietveld fit, it is difficult to judge between models A and B, and even models C and D cannot be fully excluded. The final crystallographic data of all models A–D are given in Table 3 ▸.

The powder pattern shows several reflections which have a much larger peak width than the others. In the unit cell setting of model B, this includes the reflections 102 (at 2θ = 15.9°) and 302 (at 2θ = 20.8°, see Fig. 2 ▸), and – as far as visible – several other reflections (702, 104, 104 and 504). In contrast, 002, 111, 210 and all *h*00 reflections are sharp. Hence, the peak broadening is apparently not caused by anisotropic domain sizes. The peak widths could be attributed to diffuse scattering arising from some disorder, but the powder data did not allow the extraction of more information.

### 3.4. Why are the simulated powder patterns so similar?

Why are the powder patterns of the four structural models so similar, yet the structures differ strongly in their lattice parameters, space groups and their molecular packing?

In general, problems arise if the pattern is a dominant-zone pattern, *i.e.* if most or all reflections can be indexed with two Miller indices only, *e.g.* by *h*0*l*. In this case, information on the third spatial direction is missing. Correspondingly, the indexing is uncertain or even impossible, and structure solution and refinement are difficult too, because of the lacking information on the third coordinate. Examples include the structures of a methyl derivative of Pigment Red 170, which was finally solved by crystal structure prediction (Schmidt *et al.*, 2006 ▸), and of copper perchloro­phthalocyanine, which was solved by electron diffraction (Gorelik *et al.*, 2021 ▸). However, DFQ does not exhibit a dominant-zone pattern. All reflections at 2θ < 23° are *h*0*l* reflections (*hk*0 for model C), but the reflections at 2θ > 23° are mostly mixed *hkl* reflections, which provide more than sufficient information on the third spatial direction (see Fig. S3). Hence, dominant-zone can be ruled out as an explanation for the similarity of the powder patterns.

**Figure 6:**
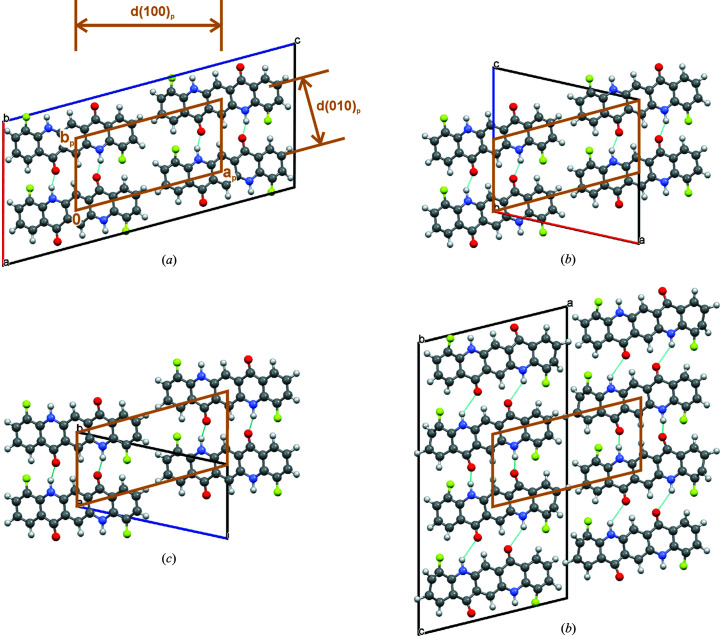
Structures A–D. Arrangement of the molecular stacks. Viewed along the stacking direction. The brown parallelogram marks the unit cell of the two-dimensional parallelogram pattern formed by the stacks, regardless of the orientation of the molecules within the stacks. This unit cell is similar for all structures. The net plane distances *d*(100)_p_ and *d*(010)_p_ of this unit cell are also similar.

In fact, the reason the simulated patterns are so similar is that the crystal structures have many features in common:

(*a*) All molecules are close to planar.

(*b*) In all structures the molecules are stacked on top of each other, thereby forming linear stacks (columns).

(*c*) Within the stacks, the distance between the mean molecular planes is very similar: 3.445 to 3.483 Å, corresponding to the thickness of the molecules (see Table 4 ▸).

(*d*) In all structures, the molecules are not perpendicular to the stacking direction, but form an inclination angle τ of about 66° with the stacking direction. This inclination angle is similar for all structures, see Table 4 ▸.

(*e*) In all structures the stacks are parallel.

(*f*) In all structures the stacks arrange in an oblique two-dimensional parallelogram pattern (see Fig. 6 ▸).

(*g*) The lateral dimensions of the stacks are determined by the lengths and widths of the molecule. Correspondingly, the dimensions *a*_p_, *b*_p_ and γ_p_ of the parallelogram pattern are quite similar for all structures, see Fig. 6 ▸ and Table 4 ▸.

(*h*) Accordingly, the corresponding net plane (100)_p_ of the parallelogram pattern has a fairly similar distance of *d*(100)_p_ ≃ 13.9 Å in all structures. The same is true for the net plane (010)_p_ with *d*(010)_p_ ≃ 6.6 Å (see Fig. 6 ▸).

(*i*) In all structures, the net plane distance *d*(100)_p_ corresponds to the first reflection in the powder pattern at 2θ = 6.35°. Since the unit cell settings of models A–D are different, the *hkl* indices in the individual models are different. It is the 002 reflection in model A, the 100 in models B and D, and the 001 in model C. The second reflection at 2θ = 12.76° is the second-order diffraction of the first reflection.

(*j*) The distance *d*(010)_p_ corresponds to the third peak in the powder diagram, with a 2θ value of 13.32°.

(*k*) The strong reflection at 2θ = 26.9° reflects the interplanar distance between the molecular planes, *i.e.* the thickness of the molecules. This is the 014 reflection in model A, the 210 in model B, the 102 in model C and the 212 in model D.

These similarities explain why all models lead to very similar powder patterns, although the molecular packing and lattice parameters, as well as the indexing of the individual reflections, are different.

### 3.5. *CheckCIF* test

Nowadays, every crystal structure deposited in the CSD (Allen, 2002 ▸) should pass the *checkCIF* test. *CheckCIF* consists of a huge assembly of tests for reliable molecular geometries, intermolecular contacts, plausible displacement parameters, residual electron density, missed symmetry, unconventional unit-cell settings, *etc*. (https://checkcif.iucr.org; Spek, 2020 ▸). Structures determined from powder data of limited quality usually cause several *checkCIF* alerts, *e.g.* on unusual bond lengths, low bond precision and residual electron density. Apart from these ‘usual’ alerts, the Rietveld-refined structures of A–D passed the *checkCIF* tests with only a few alerts. The only significant one was a warning that model A contains a B-centering as pseudo-symmetry element. This centering corresponds to a unit-cell transformation from model A to model B.

Apart from this justified warning on model A, each of the structures A–D could have been published, though at least two of them were completely wrong.

### 3.6. Refinement of the crystal structures by fit to the pair distribution function

A fit to the PDF is a very new method in structure refinement of organic compounds (Prill *et al.*, 2016 ▸; Schlesinger *et al.*, 2021 ▸). It has shown its success, for example, in the refinement of the disordered structure of 2-methyl­quinacridone (Schlesinger *et al.*, 2020 ▸).

The PDF is the Fourier transform of the powder pattern. Hence, the PDF contains in principle the same amount of information as the powder pattern itself. However, the Bragg reflections in the powder pattern reflect the average long-range order, whereas the PDF curve describes the local ordering. Hence, the PDF is – like SSNMR – a good method to add insight into the local structure.

**Figure 7:**
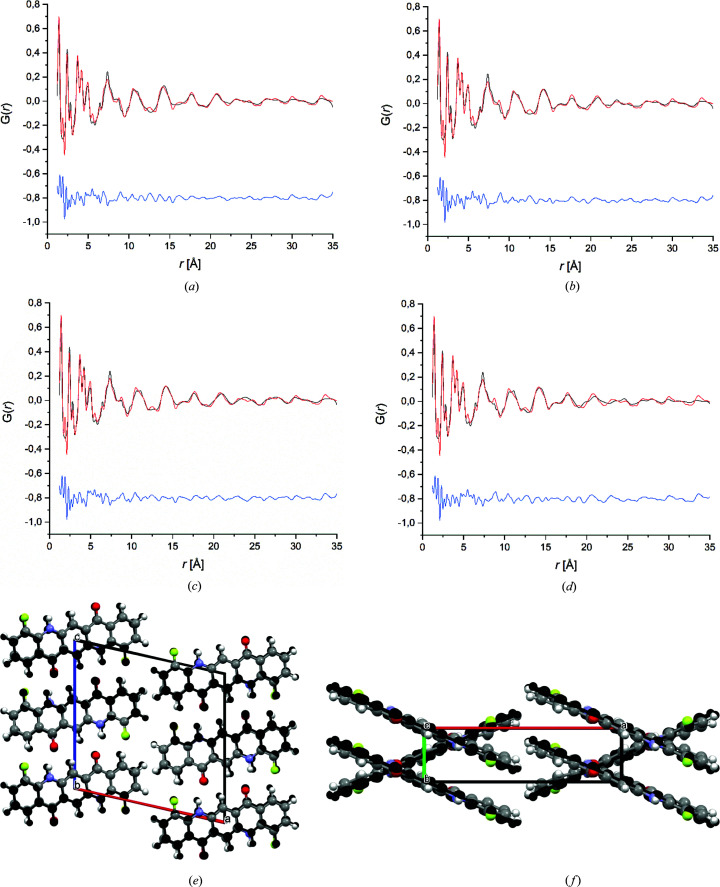
Crystal structure refinement of models A–D by a fit to the PDF using the rigid-body approach with the room-temperature data: (*a*) A, (*b*) B, (*c*) C and (*d*) D. Observed PDF is shown by the red line, calculated PDF is shown by the black line and the difference curve below is blue. (*e*) and (*f*) Comparison of model B obtained by Rietveld refinement (black) and by fit to the PDF (coloured). View directions: (*e*) [010], (*f*) [001].

The structural models were fitted to the PDF using the synchrotron data collected at room temperature and at 173 K. The molecular geometry was described either as a rigid body using the *z*-matrix algorithm or by refining all atomic coordinates using restraints on bond lengths and bond angles. All refinements converged with a good fit to the PDF curve and quite smooth difference curves (see Figs. 7 ▸ and S4–S6). An overview on all refinements is shown in Table 5 ▸. At first glance, the *R*-values seem to be quite high, but the 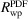 values for PDF refinements of organic compounds are generally much higher than the corresponding *R*-values for single-crystal or Rietveld refinements. For poorly crystalline organic compounds, 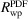 values of 20 to 30% denote a reliable fit (Prill *et al.*, 2016 ▸; Schlesinger *et al.*, 2020 ▸).

The PDF refinements with flexible molecules using restraints led to a considerable distortion of the molecules. In both approaches, the room-temperature diffraction data had a better *Q*_max_ value than the low-temperature data and allowed a better fit of the structures to the PDF. Correspondingly, the rigid-body refinements to the room-temperature PDF are discussed further.

All structural models resulted in a good fit to the PDF and in acceptable 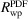 values which were very close to each other. Only the fit of model D was slightly worse. The good fit to the PDF shows that, in principle, all structural models A–D describe the average structure as well as the local structure sufficiently well.

During the PDF refinements, the structures did not change much. None of lattice parameters *a*, *b* or *c* changed by more than 0.2% from the values determined by Rietveld refinements (see Table 6 ▸). The lattice angles changed by only 0.2–0.3° in models A and B, but by 2–4° in models C and D. The latter observation is a weak indication that models A and B are a better simultaneous representative of the average structure and the local structure. For model B, the overlay of the structure from the PDF fit and from Rietveld refinement is shown in Figs. 7 ▸(*e*) and 7(*f*).

The difference curve between observed and calculated PDFs for all structural models is quite smooth in the range 1–8 Å, which corresponds to the intramolecular atom–atom distances fixed by the *z*-matrix formalism, and the closest intermolecular atom–atom distances, especially those between neighbouring and next-neighbouring molecules within the same stack (π–π stacking). Since all structures contain the same molecule, and the arrangement of the molecules within the stacks is very similar for all structures, it is evident that models A–D give quite similar PDF curves in the low-*r* region. At higher *r*, the atom–atom distances between molecules of different stacks become more frequent. Since the orientation of the molecules in neighbouring stacks differs in models A–D, the PDF curves differ more at higher *r* values.

In the room-temperature data, the 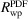 values of all four models are quite similar and do not allow the selection of the correct model. The PDF fits to the 173 K data clearly show that models A or B give a better fit than models C and D. This means that models C and D provide a poorer description of the local structure of DFQ than models A and B.

### 3.7. Evaluation of the colour

The colour of quinacridone pigments strongly depends on the arrangement of the molecules in the solid state. For most derivatives, the effect of the crystal packing appears to be even higher than the electronic effect of the substituent. Quinacridones typically form chain structures, such as model C, or criss-cross structures, such as models A and B. Chain structures result in a more bluish hue, criss-cross structures in a more yellowish hue. For example, the chain polymorphs of unsubstituted quinacridone (α^I^, α^II^ and β phases) are red to reddish violet, the chain-forming compounds 2,9-di­methyl­quinacridone and 2,9-di­chloro­quinacridone have magenta to violet shades. In contrast, the criss-cross packing of 4,11-di­chloro­quinacridone leads to a bright orange-red shade. The individual quinacridone molecule has a yellow to orange shade, which is visible in diluted solution. The reason for the colour differences between the different crystal structures is not fully understood. Obviously, not only the hydrogen-bonded network, but also exciton interactions between neighbouring molecules play a role.

**Figure 8:**
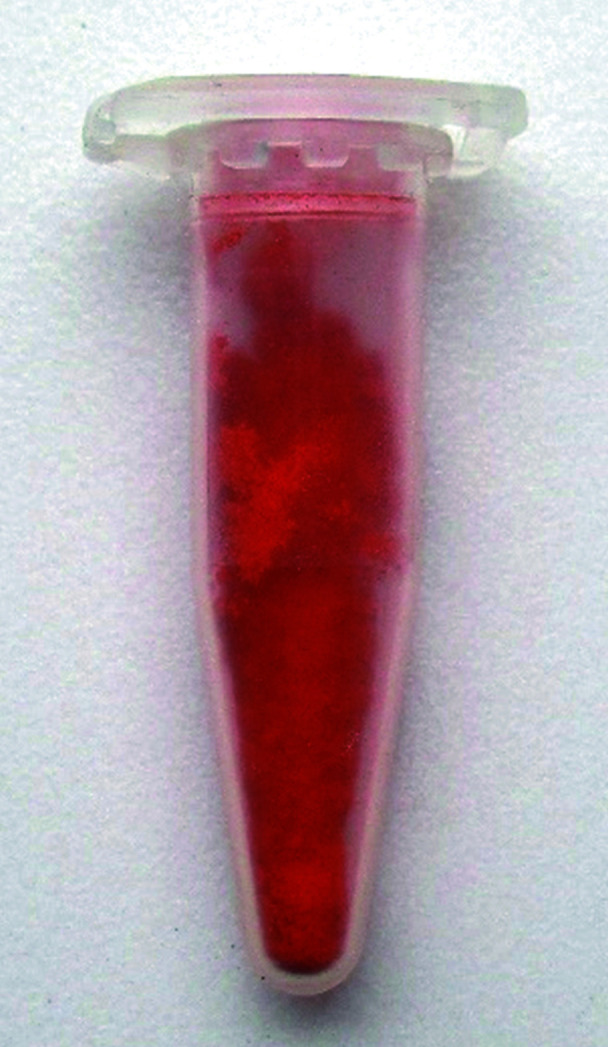
Colour of 4,11-DFQ.

DFQ has an orange shade (see Fig. 8 ▸), which points to a criss-cross packing, hence, models A or B.

### 3.8. Force-field calculations

The lattice-energy minimizations started from the structures determined by the Rietveld refinement. The results are given in Table 7 ▸. During the minimizations, several lattice parameters changed strongly (lattice constants up to 0.8 Å, lattice angles up to 11°). The best energy was achieved for model B. Models A and B did not convert to the same structure, but the small difference between the structures was maintained during the optimization. The force-field calculations indicated model B to be the correct one.

### 3.9. DFT-D calculations

#### 3.9.1. Calculations with *GRACE*

In the lattice-energy calculations with *GRACE*, using the PBE functional and the dispersion correction by Neumann & Perrin (2005 ▸), all models optimized well, and the resulting crystal structures were chemically fully sensible.

In the DFT-D optimizations with free lattice parameters, models A and B became identical, and the final structure had the symmetry of B, *i.e.* with molecules on inversion centres.

The optimization of model D with free lattice parameters led to surprising results. Two different structures were obtained, depending on whether the optimizations started from the Rietveld structure, or from the crystal structure previously optimized by force-field methods. Both structures exhibit a criss-cross packing of dimers, but they differ in their simulated powder patterns and in their lattice energies. The more favourable energy was reached starting with the structure previously optimized by force-field optimization.

The DFT-D lattice energies reveal that structures A and B are, energetically, considerably more favourable than structures C or D (see Table 8 ▸). Hence, the DFT-D lattice energies point to B as the correct solution.

Furthermore, the magnitude of structural changes during the DFT-D optimization gives an indication on the correctness of a structural model. In a large validation study, van de Streek & Neumann (2014 ▸) performed DFT-D optimizations of 215 organic crystal structures determined from powder data. The same DFT functional and dispersion correction were used for the current calculations. The lattice parameters were also optimized. van de Streek calculated the RMSCD of the non-hydrogen atoms before and after optimization (for the formula, see van de Streek & Neumann, 2014 ▸). They concluded that RMSCD values below 0.35 Å indicate that the structures are correct. Larger deviations point to errors such as wrong hydrogen atom positions or wrong atom assignment. The RMSCD values of the models of DFQ are given in Table 8 ▸. According to the RMSCD values, models A and B should be regarded as correct, whereas models C and D are likely to be incorrect.

#### 3.9.2. Calculations with *Quantum Espresso*

*Quantum Espresso* calculations were performed with a double aim: to provide a different methodology with the non-local van der Waals approach and to validate the crystal structure by comparing experimental and theoretical SSNMR chemical shifts as well as SSNMR ^13^C and ^1^H chemical shift root mean squares (RMSs).

In the geometry optimizations with *Quantum Espresso*, models A and B became identical, like in the *GRACE* calculations. Only the unit-cell setting is different (see Fig. S7).

The fixed and free cell optimizations are very similar, with the exception of model C. For this structure the difference of volume between free and fixed cell optimization is only 2.7%, but the shape of the cell is completely reorganized. This indicates, from a computational viewpoint, that the starting structure is thermodynamically unfavourable and less probable.

### 3.10. Solid-state NMR

In the lattice-energy optimizations of models A–D with *Quantum Espresso* at the vdW-DF2 level with PBE pseudopotentials, the most stable structure is model A with the relaxed cell. Models A and B (with fixed or relaxed cells) have the same lattice energy, and very similar structures, thus they can be considered equivalent. Model C is significantly higher in energy (see Table 9 ▸).

**Figure 9:**
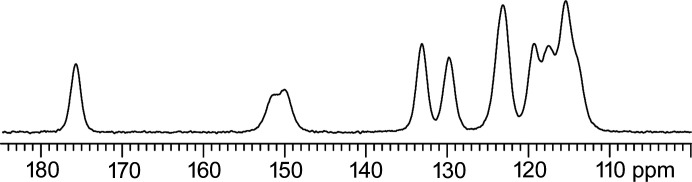
^13^C (150.9 MHz) CPMAS SSNMR spectrum of DFQ acquired at a spinning speed of 20 kHz.

The ^13^C CPMAS spectrum is shown in Fig. 9 ▸, and ^1^H and ^19^F MAS and ^19^F–^13^C CPMAS spectra are reported in Figs. S8 and S9.

The ^13^C CPMAS spectrum is characterized by broad peaks (FWHM ≃ 230 Hz) in agreement with the poor crystallinity observed in the powder diffraction (see above). Interestingly, the number of resonances suggests the presence of half a molecule per asymmetric unit, hence a structure with *Z*′ = 0.5. However, several shoulders, in particular the one at 114.1 p.p.m., indicate the presence of disorder, but impurities or more independent sites or a combination are also possible. The additional peaks (at 5.6, 4.7 and 2.2 p.p.m.) observed in the ^1^H MAS spectrum [see Fig. S9(*a*)] show the presence of impurities, but do not exclude disorder. The origin of the double peak for the CF C4/C11 sites at 150.0 and 151.3 p.p.m., whether arising from the ^1^J_CF_ or independent sites, was investigated through the ^19^F–^13^C{^19^F} CPMAS spectrum [see Fig. S8(*b*)]: here, the presence of a single signal (at 150.8 p.p.m.) confirms the former hypothesis, ruling out the presence of independent sites for C4 and C11; therefore the molecule must be situated on a special position with *Z*′ = 0.5, and C4 and C11 give rise to a single peak being equivalent. This is also supported by the ^19^F MAS spectrum [see Fig. S9(*b*)] where a single signal at −133.8 p.p.m. is observed.

This experimental evidence points toward model B or C and against models A and D, since the number of experimental signals is lower than those expected for these structures.

As a further variable independent of the energy and the fit to the powder pattern and the PDF, we also considered the calculated chemical shifts and the relative RMS values (see Table S1 in the supporting information) with respect to the experimental ones. The combination of these two parameters can be used for a more reliable selection of the best structure. Although it has been proven that the comparison of ^1^H chemical shifts is the most reliable (Salager *et al.*, 2010 ▸), in this case the ^13^C chemical shifts were also compared since the proton spectrum is characterized by only two signals (NH at 11.5 p.p.m. and CH at 7.6 p.p.m.) attributed to DFQ. The comparison of the ^1^H MAS and ^13^C CPMAS experimental spectra with the computed ones for the four models are reported in Figs. S10 and S11 while the RMS values are shown in Table 10 ▸. The small difference in the ^1^H and ^13^C RMS values observed for the four models suggests that, even if the packing is different, the local structure (H bond model, …) is very similar, near the distinction limit of the approach. However, significant differences are observed for some of the computed resonances, for instance, the ^1^H NH (calc. 9.52 p.p.m. versus exp. 11.5 p.p.m.) and the ^13^C C10 (calc. 111.93 p.p.m. versus exp. 115.4 p.p.m.) chemical shifts for models C and D, respectively.

In any case, even considering the limits of the method, both ^1^H and ^13^C RMS values agree with the conclusions drawn from the XRD and the calculation data: models A and B can be considered equivalent and fit better with the experimental data than C and D. Thus, by further considering the *Z*′ value, B seems to be the best model for describing the structure of DFQ.

## 4. Discussion

### 4.1. Which is the correct structure?

From a crystallochemical point of view, all four structures A–D are fully plausible. They exhibit reliable molecular geometries, reliable hydrogen-bond patterns and molecular packings, which are observed in other quinacridone compounds as well. There are no unreliably close intermolecular contacts, and the density corresponds exactly to the expected value. All structures give a good fit to the powder data with a smooth difference curve and acceptable *R*-values. The structural models A–D even pass the *checkCIF* procedure without problems. *CheckCIF* only warns that model A actually has a higher symmetry. Hence, we could probably have published models B, C or D without problems as the crystal structure of DFQ.

Additionally, all structures give a reliable fit to the PDF data, behave well in the lattice-energy minimizations with DFT-D and exhibit calculated ^13^C chemical shifts close to the experimental values. However, only one of these structures can be correct; the other ones must be wrong or, at least, incorrect.

In the careful Rietveld refinements, models A and B show a better fit than models C and D. The same effect is seen in the PDF fits to the 173 K data. Hence, models A and B describe the average structure as well as the local structure better than models C and D. Furthermore, during the lattice-energy optimizations with DFT-D, the Rietveld-refined structures of C and D changed by more than 0.35 Å, which indicates that these structures are probably incorrect.

Additionally, in all DFT and force-field optimizations the lattice energies of C and D are significantly worse than those of A and B. Non-local van der Waals periodic calculations with *Quantum Espresso* on model C show a significant reorganization of the crystal and display the worst RMS values. Thus, model C can be ruled out from the possibilities.

SSNMR investigations highlight the presence of molecules on a special position with *Z*′ = 0.5, ruling out model D. As mentioned before, models A and B are computationally almost equivalent, but they are not equivalent from an SSNMR viewpoint because of symmetry. Furthermore, the calculated ^13^C chemical shifts of models A and B match the experimental values better than those of C and D. Thus, all experimental evidence indicates that models A or B should be correct, and C and D are wrong.

But which model is correct, A or B?

The Rietveld refinements do not give a clear answer. Model A provides a better fit to the experimental data, with a smoother difference curve and considerably lower *R*-values, but at the expense of double the number of structural parameters (*Z*′ = 1 instead of *Z*′ = 0.5).

Models A and B are different in the *FIDEL-GO* fits and in the force-field optimizations, whereas they are almost identical in the Rietveld refinements, the PDF fits and the lattice-energy minimizations with both DFT methods. In such a case, the higher symmetrical model should be chosen, *i.e.* model B with *Z*′ = 0.5, which is in agreement with the results of the SSNMR investigations. Therefore, model B should be considered the correct structural model. Additionally, the presence of broad reflections in the powder pattern indicates the compound exhibits some disorder, but details of the disorder could not be eludicated from all of our data.

### 4.2. Why do we not know more cases of ambiguous structure determinations?

Why are not more cases known in the literature, where the structure solution of an organic compound is so ambiguous that it results in structural models with strikingly different molecular arrangements, all fitting the experimental data?

There are four main reasons: (1) most crystal structures are determined from single-crystal data; the information content is so high that the molecular packing is generally correct, even if some features of the structure are incorrect (*e.g.* atom assignment, configuration at C atoms, orientation of side groups, hydrogen-bond network *etc*.). (2) Structure solutions from powder data are generally only performed if the unit-cell parameters are known, which restricts the variety of possible packing motifs (if the unit-cell parameters of DFQ were known in advance, we would have obtained only one solution). (3) Without prior knowledge of lattice parameters, crystal structures can be solved by a global crystal structure prediction. In this case, the energy is an additional variable, which helps in the selection of possible structural models. (4) Ambiguous structure solutions are rarely published: authors need to be confident and gather enough evidence that the published structure solution is correct, otherwise the structure will probably never be published.

Nevertheless, there are cases in which two structural models have similar lattice energies and both fit quite well to the diffraction data. Such a case was observed for aspirin (acetyl­salicylic acid). There, both models were actually correct: there are two polymorphs, which have similar lattice energies and similar single-crystal diffraction patterns (Bond *et al.*, 2007*a* ▸,*b* ▸).

For DFQ the structure was solved only by a fit to a low-quality powder pattern, without taking additional information into account (except for the molecular geometry). In such a case, it is no wonder that multiple structure solutions can occur. At a conference, a colleague spontaneously commented: ‘Das war zu erwarten’ [That was to be expected (Hofmann, 2018 ▸)]. However, it is surprising that all four structural models were also chemically sensible, provided a good fit to the PDF and had reasonable lattice energies and SSNMR spectra.

## 5. Conclusions

This paper once again shows that a high level of critical judgement of the results obtained from every structure determination must be applied. The mere existence of a plausible crystal structure, a good Rietveld fit with a smooth difference plot, acceptable *R*-values and a successful *checkCIF* test does not justify the attribute ‘correct structure’.

In the case of DFQ, six highly complementary methods were applied: (1) Rietveld refinements, (2) fit to the PDF, (3) evaluation of the colour, (4) lattice-energy minimizations with force fields, (5) two different DFT-D methods and (6) a multinuclear SSNMR approach (^1^H, ^19^F and ^13^C), which allowed unequivocal identification of the symmetry of the molecule and thus *Z*′ = 0.5. Only the combination of these methods allows the selection of the most reliable model.

Furthermore, this work is one of the first examples in which a fit of a crystal structure to the PDF was used in the determination of a hitherto unknown crystal structure of an organic compound.

## Supplementary Material

Crystal structure: contains datablock(s) global, DFQ_model_A_Rietveld, DFQ_model_B_Rietveld, DFQ_model_C_Rietveld, DFQ_model_D_Rietveld, DFQ_model_B_PDF-fit. DOI: 10.1107/S2052252522004237/lt5047sup1.cif

Rietveld powder data: contains datablock(s) DFQ_model_A_Rietveld. DOI: 10.1107/S2052252522004237/lt5047DFQ_model_A_Rietveldsup2.rtv

Rietveld powder data: contains datablock(s) DFQ_model_B_Rietveld. DOI: 10.1107/S2052252522004237/lt5047DFQ_model_B_Rietveldsup3.rtv

Rietveld powder data: contains datablock(s) DFQ_model_C_Rietveld. DOI: 10.1107/S2052252522004237/lt5047DFQ_model_C_Rietveldsup4.rtv

Rietveld powder data: contains datablock(s) DFQ_model_D_Rietveld. DOI: 10.1107/S2052252522004237/lt5047DFQ_model_D_Rietveldsup5.rtv

Rietveld powder data: contains datablock(s) DFQ_model_B_PDF-fit. DOI: 10.1107/S2052252522004237/lt5047DFQ_model_B_PDF-fitsup6.rtv

Supporting figures and table. DOI: 10.1107/S2052252522004237/lt5047sup7.pdf

CCDC references: 2124726, 2124727, 2124728, 2124729, 2124730

## Figures and Tables

**Table 1:** Result of the structure solution by a global fit with *FIDEL-GO. S* ^0^_12_ denotes the similarity measure after structure solution For details on *S* ^0^_12_ see Habermehl *et al.* (2022 ▸). Additionally, the result of the automated Rietveld refinement is shown

Model	*FIDEL-GO* fit		Automated Rietveld refinement								
	Rank	*S* ^0^_12_	Rank	*R*_wp_ (%)	*a* (Å)	*b* (Å)	*c* (Å)	α (°)	β (°)	γ (°)	Vol (Å^3^)
A	1	0.9891	2	10.888	13.687	3.770	28.687	90	104.54	90	1432.93
B	2	0.9875	1	7.484	14.391	3.763	13.603	90	105.33	90.	710.50
C	3	0.9840	3	11.966	3.896	7.043	14.201	102.26	86.80	105.06	367.71
D	4	0.9819	4	12.822	14.148	3.764	27.144	90	101.66	90	1415.75

**Table 2:** Results of the user-controlled Rietveld refinements of models A–D of DFQ *R*_wp_′ denotes the background-corrected *R*_wp_ value

Model	SG (*Z*′)	Rank	*R*_exp_	*R*_wp_′	GoF	*a* (Å)	*b* (Å)	*c* (Å)	α (°)	β (°)	γ (°)
A	*P*2_1_/*c* (1)	1	1.276	9.018	4.045	13.6961 (12)	3.76836 (13)	28.789 (3)	90	105.163 (12)	90
A†	*P*2_1_/*c* (0.5)					14.2314 (16)	3.76836 (13)	13.6961 (12)	90	102.511 (12)	90
B	*P*2_1_/*c* (0.5)	2	1.280	12.249	5.279	14.2172 (15)	3.76778 (16)	13.7038 (13)	90	102.504 (12)	90
C	*P* 1 (0.5)	4	1.280	17.769	7.770	3.8854 (5)	7.0333 (10)	14.101 (3)	102.713 (16)	86.084 (14)	105.939 (16)
D	*P*2_1_/*c* (1)	3	1.276	14.796	6.718	14.335 (2)	3.7727 (3)	27.374 (4)	90	103.69 (2)	90

† After unit cell transformation with *a*′ = (−*c* − *a*)**/**2, *b*′ = *b*, *c*′ = *a*.

**Table 3:** Final crystallographic data of the four structural models A–D of DFQ from the Rietveld refinement The last column provides the data of model B from the fit to the PDF at 298 K using a rigid molecule

	Model A	Model B	Model C	Model D	Model B, fit to the PDF
Structure from	Rietveld ref.	Rietveld ref.	Rietveld ref.	Rietveld ref.	Fit to the PDF
Crystal data					
Chemical formula	C_20_H_10_F_2_N_2_O_2_	C_20_H_10_F_2_N_2_O_2_	C_20_H_10_F_2_N_2_O_2_	C_20_H_10_F_2_N_2_O_2_	C_20_H_10_F_2_N_2_O_2_
CCDC number	2124726	2124727	2124728	2124729	2124730
*M*_r_	348.30	348.30	348.30	348.30	348.30
Crystal system	Monoclinic	Monoclinic	Triclinic	Monoclinic	Monoclinic
Space group (No.)	*P*2_1_/*c* (14)	*P*2_1_/*c* (14)	*P* 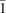 (2)	*P*2_1_/*c* (14)	*P*2_1_/*c* (14)
*Z*, *Z*′	4, 1	2, 1/2	1, 1/2	4, 1	2, 1/2
Temperature (K)	298	298	298	298	298
*a* (Å)	13.6961 (12)	14.2172 (15)	3.8854 (5)	14.335 (2)	14.166 (17)
*b* (Å)	3.76836 (13)	3.76778 (16)	7.0333 (10)	3.7727 (3)	3.758 (3)
*c* (Å)	28.789 (3)	13.7038 (13)	14.101 (3)	27.374 (4)	13.762 (14)
α (°)	90	90	102.713 (16)	90	90
β (°)	105.163 (12)	102.504 (12)	86.084 (14)	103.69 (2)	103.09 (15)
γ (°)	90	90	105.939 (16)	90	90
*V* (Å^3^)	1434.1 (2)	716.67 (11)	361.44 (11)	1438.4 (4)	713.5 (13)
ρ_calc_ (10^3^ kg m^−3^)	1.613	1.614	1.600	1.608	1.621
Radiation type	Cu *K*α_1_	Cu *K*α_1_	Cu *K*α_1_	Cu *K*α_1_	Synchrotron
Wavelength (Å)	1.5406	1.5406	1.5406	1.5406	0.1631
μ (mm^−1^)	1.047	1.048	1.039	1.044	0.028

Data collection					
Diffractometer	Stoe Stadi-P	Stoe Stadi-P	Stoe Stadi-P	Stoe Stadi-P	Synchrotron
Specimen mounting	Polymer films	Polymer films	Polymer films	Polymer films	Capillary
Data collection mode	Transmission	Transmission	Transmission	Transmission	Transmission
Detector	Linear position-sensitive detector	Linear position-sensitive detector	Linear position-sensitive detector	Linear position-sensitive detector	Perkin-–Elmer area detector
2θ_min_ (°)	4.5	4.5	4.5	4.5	–
2θ_max_ (°)	80	80	80	80	–
2θ_step_ (°)	0.01	0.01	0.01	0.01	–

Refinement					
*R*_p_ (%)	3.875	4.912	7.488	6.395	
*R*_wp_ (%)	5.160	6.754	9.944	8.657	
*R*_exp_ (%)	1.276	1.280	1.280	1.276	
*R*_p_′ (%)†	8.005	10.919	16.099	12.746	
*R*_wp_′ (%)†	9.019	12.239	17.676	14.946	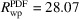
*R*_exp_′ (%)†	2.230	2.320	2.275	2.203	
GoF	4.045	5.275	7.768	6.786	
No. of data points	7550	7550	7550	7550	
No. of parameters	158	104	118	158	
No. of restraints	69	61	60	69	
Hydrogen atom treatment	Refined with restraints	Refined with restraints	Refined with restraints	Refined with restraints	Rigid molecule

† Values are background-corrected data according to Coelho (2004 ▸).

**Table 4:** Common features of models A–D *d*_m_ denotes the distance between the mean molecular plane of neighbouring molecules within a stack. τ is the inclination angle between the mean molecular plane and the stacking direction. *a*_p_, *b*_p_ and γ_p_ are the dimensions of the two-dimensional parallelogram pattern formed by the stacks, regardless of the orientation of the molecules within the stacks. *d*(100)_p_ and *d*(010)_p_ are the corresponding net plane distances

Model	*d*_m_ (Å)	τ (°)	*a*_p_ (Å)	*b*_p_ (Å)	γ_p_ (°)	*d*(100)_p_ (Å)	*d*(010)_p_ (Å)
A	3.460	66.67	14.394	6.348	74.84	13.894	6.610
B	3.445	66.10	14.384	6.852	74.79	13.880	6.612
C	3.483	63.71	14.068	6.763	77.89	13.755	6.612
D	3.462	66.61	13.339	6.846	76.19	13.924	6.648

**Table 5:** Structure refinement of models A–D by fit to the PDF, using different datasets and refinement procedures

		Refinement with rigid molecules using the *z*-matrix formalism		Refinement with flexible molecules using restraints	
Model	SG (*Z*′)	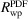 (%)	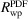 (%)	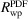 (%)	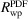 (%)
*T* (K)		298	173	298	173
A	*P*2_1_/*c* (1)	28.10	30.47	24.05	25.34
B	*P*2_1_/*c* (0.5)	28.07	30.26	24.13	25.71
C	*P* 1 (0.5)	28.09	32.59	24.14	28.49
D	*P*2_1_/*c* (1)	29.01	32.61	25.50	28.46

**Table 6:** Crystal structural models obtained by fit to the PDF (298 K data, rigid molecules)

Model	SG (*Z*′)	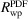	*a* (Å)	*b* (Å)	*c* (Å)	α (°)	β (°)	γ (°)
A	*P*2_1_/*c* (1)	28.10	13.670	3.762	28.690	90	105.473	90
A†	*P*2_1_/*c* (0.5)		14.149	3.762	13.670	90	102.278	90
B	*P*2_1_/*c* (0.5)	28.07	14.166	3.758	13.762	90	103.088	90
C	*P* 1 (0.5)	28.09	3.857	7.080	13.916	102.559	86.508	103.672
D	*P*2_1_/*c* (1)	29.01	14.286	3.756	27.701	90	107.174	90

† Unit-cell parameters after transformation with *a*′ = (−*c* − *a*)/2, *b*′ = *b*, *c*′ = *a*.

**Table 7:** Results of the force-field optimizations of the four structural models, including optimization of the lattice parameters *E*_rel_ denotes the lattice energy (per molecule) relative to model B

Model	SG (*Z*′)	*E*_rel_ (kJ mol^−1^)	*a* (Å)	*b* (Å)	*c* (Å)	α (°)	β (°)	γ (°)
A	*P*2_1_/*c* (1)	7.66	14.01	3.80	28.98	90	95.65	90
A†	*P*2_1_/*n* (1)		30.92	3.80	14.01	90	111.15	90
B	*P*2_1_/*c* (0.5)	0	13.93	3.98	13.79	90	98.59	90
C	*P* 1 (0.5)	9.71	3.88	6.64	14.88	97.99	78.13	94.30
D	*P*2_1_/*c* (1)	13.81	14.69	3.73	27.48	90	100.15	90

† After unit transformation with *a*′ = −*c* − *a*, *b*′ = *b*, *c*′ = *a*.

**Table 8:** Results of the DFT-D optimizations of the four structural models with *GRACE*, including optimization of the lattice parameters RMSCD is the root mean-square Cartesian deviation of the non-hydrogen atoms between the structure from Rietveld refinement and from DFT-D optimization. *E*_rel_ denotes the lattice energy (per molecule) relative to model A. Models A and B are identical, except for a different unit-cell setting

Model	SG (*Z*′)	RMSCD (Å)	*E*_rel_ (kJ mol^−1^)	*a* (Å)	*b* (Å)	*c* (Å)	α (°)	β (°)	γ (°)
A	*P*2_1_/*c* (1)	0.31	0	13.477	3.832	29.002	90	107.93	90
A†	*P*2_1_/*c* (0.5)			13.982	3.832	13.477	90	99.36	90
B	*P*2_1_/*c* (0.5)	0.27	+0.07	14.011	3.830	13.483	90	99.68	90
C	*P* 1 (0.5)	0.51	+13.00	4.677	7.098	13.840	109.64	84.87	124.60
D (start: RV) ‡	*P*2_1_/*c* (1)	0.87	+10.94	14.295	3.861	26.481	90	98.68	90
D (start: FF) §	*P*2_1_/*c* (1)	0.79	+7.71	14.467	3.806	26.913	90	106.10	90

† Lattice parameters after unit transformation with *a*′ = (−*c* − *a*)/2, *b*′ = *b*, *c*′ = *a*.

‡ Optimization starting from the Rietveld-refined structure.

§ Optimization starting from the crystal structure optimized by force-field methods.

**Table 9:** Structural parameters of models A–D optimized by *Quantum Espresso*; energies are relative to model A (relaxed cell)

Model	SG (*Z*′)	*E*_rel_ (kJ mol^−1^)	*a* (Å)	*b* (Å)	*c* (Å)	α (°)	β (°)	γ (°)	*V* (Å^3^)
A†	*P*2_1_/*c* (0.5)	1.6	13.668	3.766	34.079	90	125.53	90	1427.72
A		0.00	13.583	3.658	33.598	90	124.55	90	1374.96
B†	*P*2_1_/*c* (0.5)	1.6	14.217	3.768	13.704	90	102.50	90	716.67
B		0.02	14.255	3.663	13.570	90	102.47	90	691.90
C†	*P* 1 (0.5)	33.0	3.885	7.033	14.101	102.71	86.08	105.94	361.44
C		20.2	3.709	6.512	14.891	80.54	91.37	82.88	351.63
D†	*P*2_1_/*c* (1)	14.1	14.339	3.772	27.385	90	103.81	90	1438.46
D		13.1	14.392	3.672	27.204	90	103.64	90	1396.92

† Fixed cell parameters, obtained from preliminary Rietveld refinements

**Table 10:** ^1^H and ^13^C SSNMR RMS deviation between calculated and experimental chemical shifts (p.p.m.) for structures A–D For the calculation of the SSNMR shifts, the crystal structures were optimized with a fixed unit cell.

Model	SG (*Z*′)	^1^H RMS	^13^C RMS
A	*P*2_1_/*c* (1.0)	0.82	2.28
B	*P*2_1_/*c* (0.5)	0.79	2.29
C	*P* 1 (0.5)	1.16	2.37
D	*P*2_1_/*c* (1)	0.85	2.39
