# Supplementary material for: Ambiguous structure determination from powder data: four different structural models of 4,11-di­fluoro­quinacridone with similar X-ray powder patterns, fit to the PDF, SSNMR and DFT-D
Source: IUCrJ. 2022 May 14;9(Pt 4):406–24. doi: 10.1107/S2052252522004237 (PMC9252154; doi:10.1107/S2052252522004237)
Supplement: Supplementary file 7 [file m-09-00406-sup7.pdf]

IUCrJ

Volume 9 (2022)

Supporting information for article:

Ambiguous structure determination from powder data: Four different structural models of 4,11-difluoroquinacridone with similar X-ray powder patterns, fit to the PDF, SSNMR and DFT-D

Carina Schlesinger, Arnd Fitterer, Christian Buchsbaum, Stefan Habermehl, Michele R. Chierotti, Carlo Nervi and Martin U. Schmidt

Supporting Information
for
"Ambiguous structure determination from powder data: Four different structural models of 4,11-difluoroquinacridone with similar X-ray powder patterns, fit to the PDF, SSNMR and DFT-D"

by

Carina Schlesinger, Arnd Fitterer, Christian Buchsbaum, Stefan Habermehl, Michele R. Chierotti, Carlo Nervi and Martin U. Schmidt

Results of the structure solution of 4,11-difluorquinacridone by a global fit to the powder data with FIDEL

a)

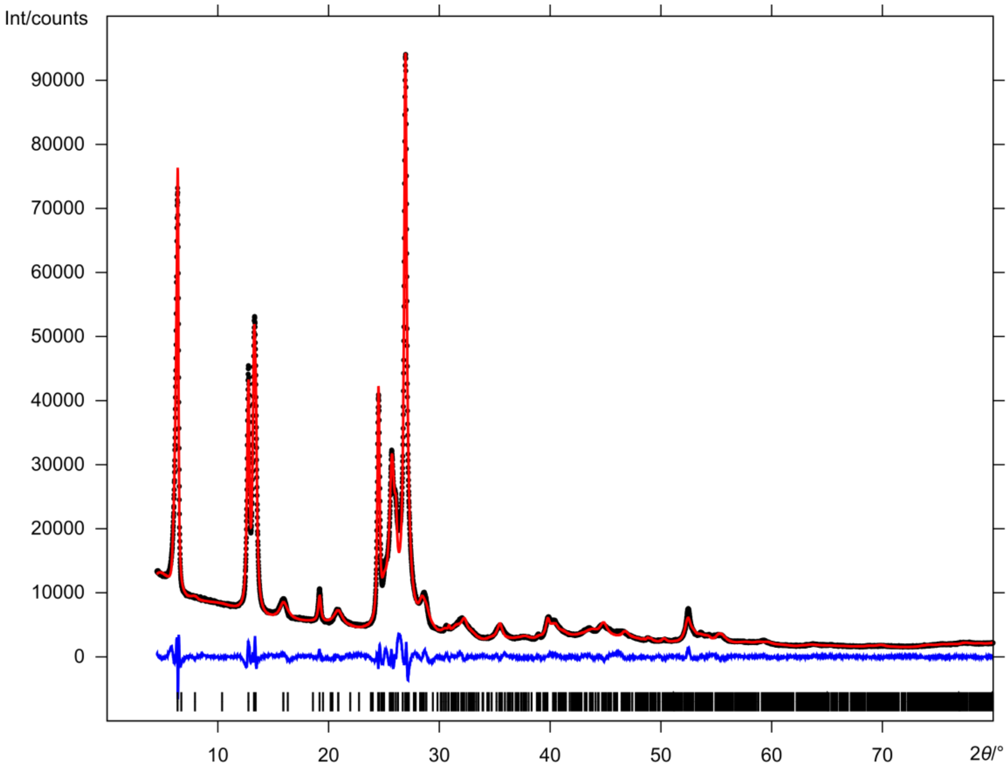

b)

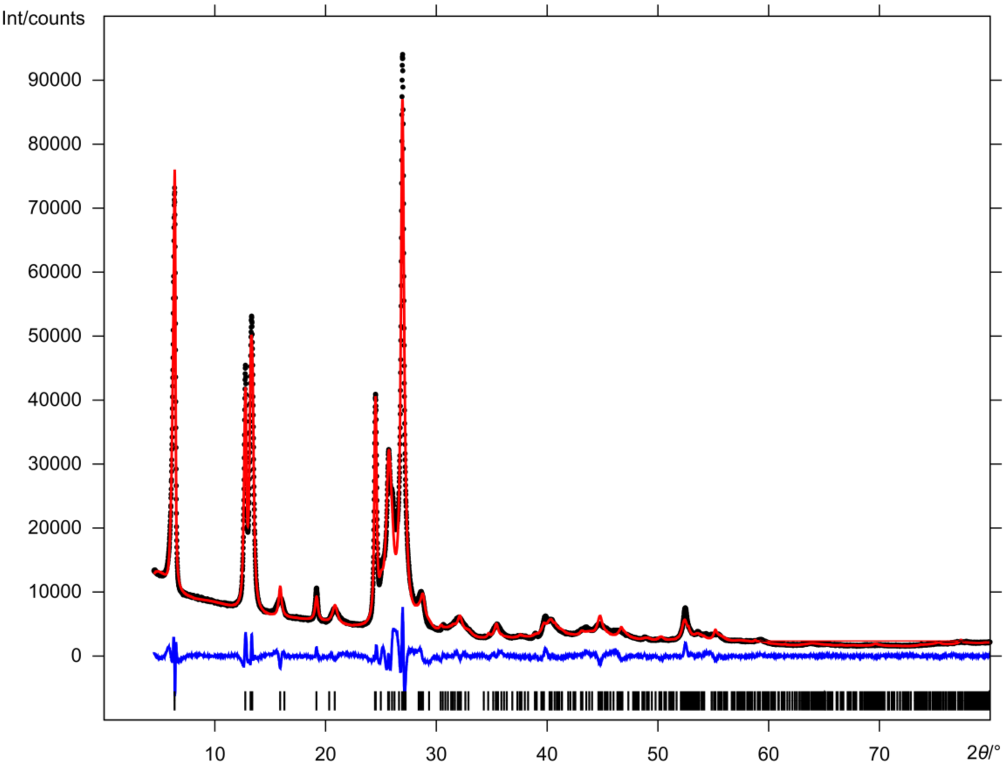
Int/counts
90000
80000
70000
60000
50000
40000
30000
20000
10000
0
10
20
30
40
50
60
70
2θ/°

c)

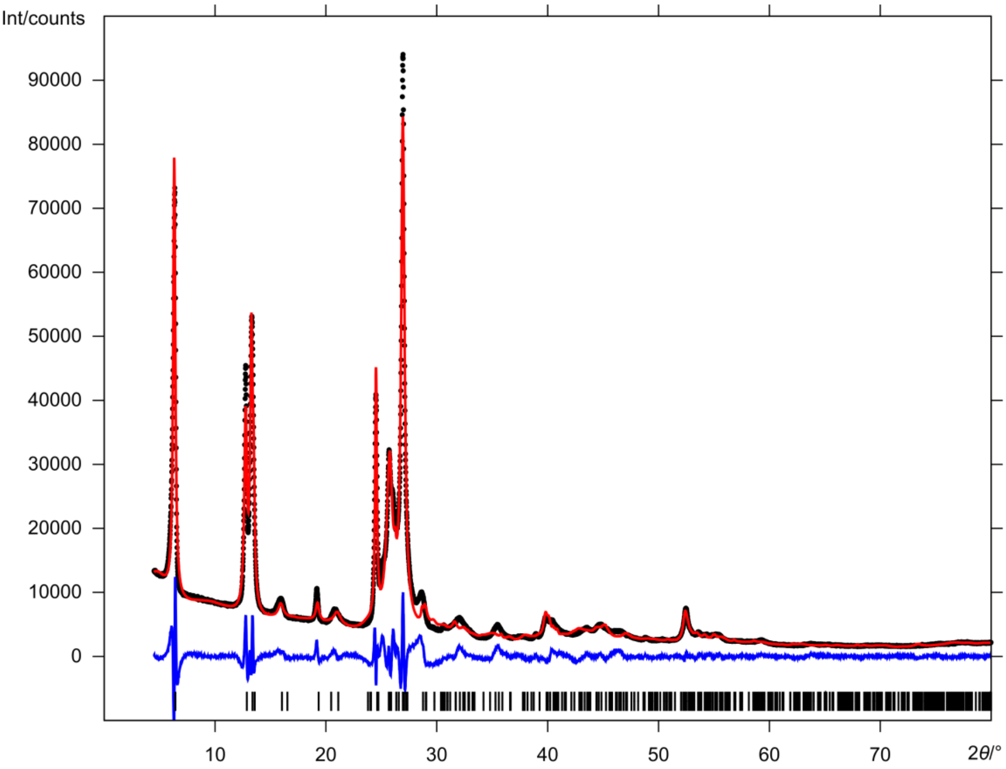
Int/counts
90000
80000
70000
60000
50000
40000
30000
20000
10000
0
10
20
30
40
50
60
70
2θ/°

d)

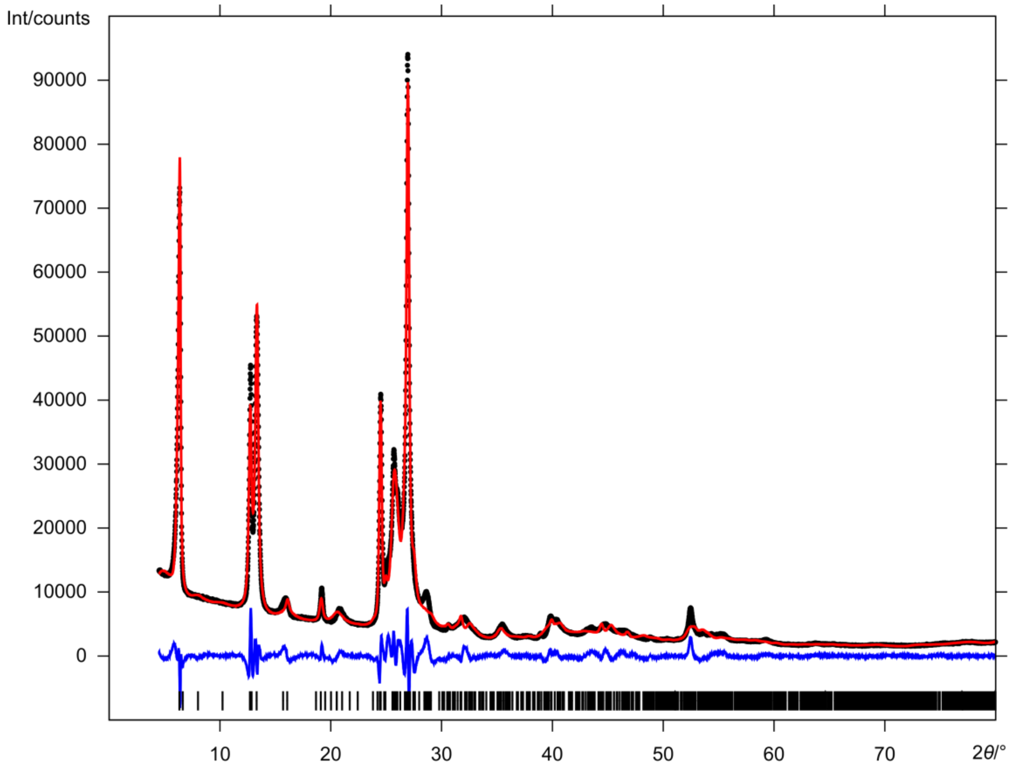

Figure S1. Rietveld plots of the automated Rietveld refinements of 4,11-difluorquinacridone. (a) model A, (b) B, (c) C (d) D. Observed intensities are denoted as black dots; calculated intensities as a red line, difference curve below in blue, vertical ticks represent the reflection positions.

Pawley fits

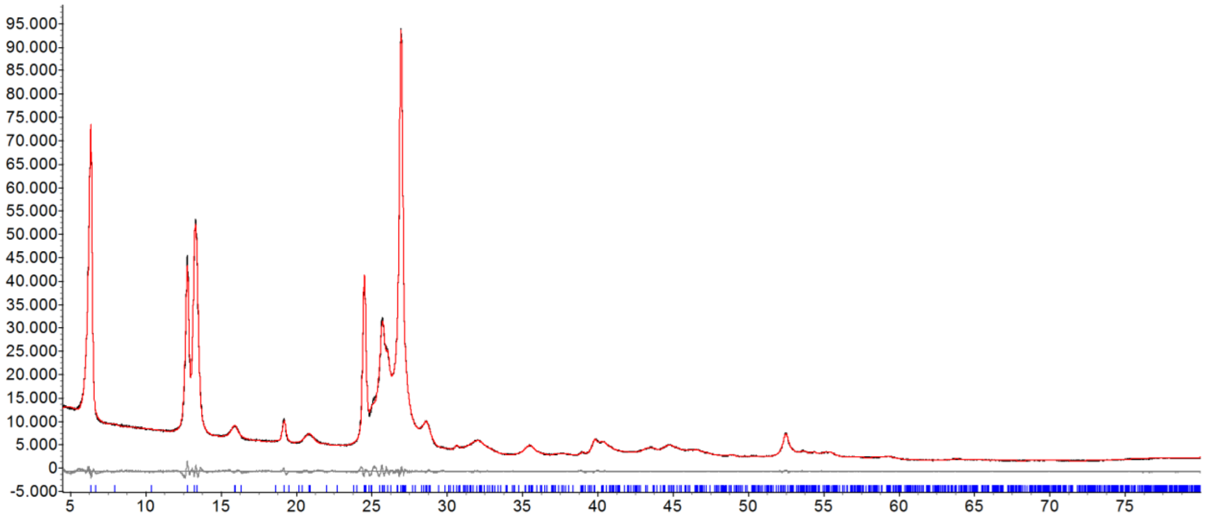

Model A, Pawley fit

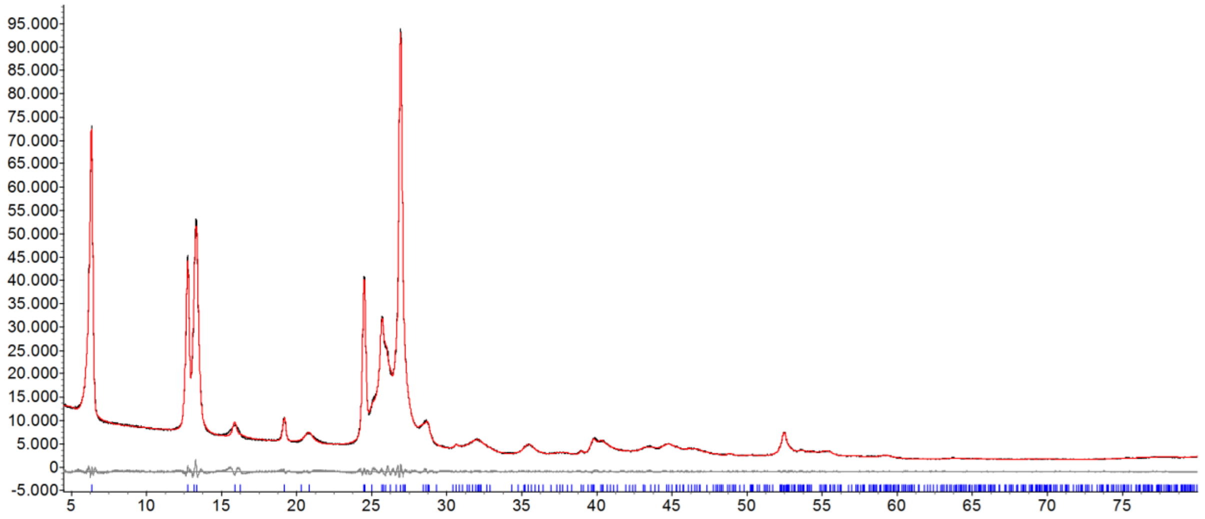

Model B, Pawley fit

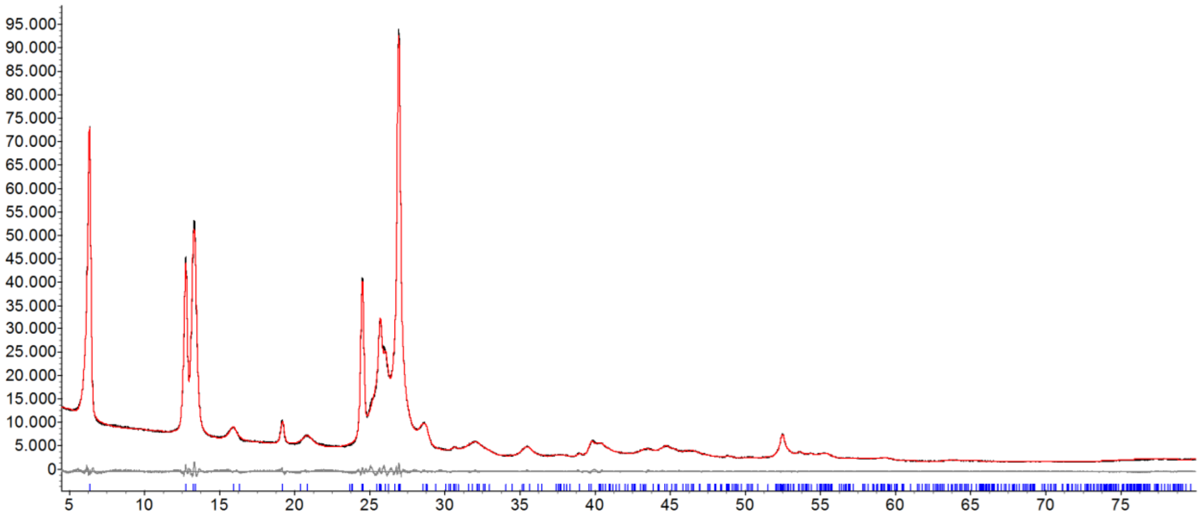

Model C, Pawley fit

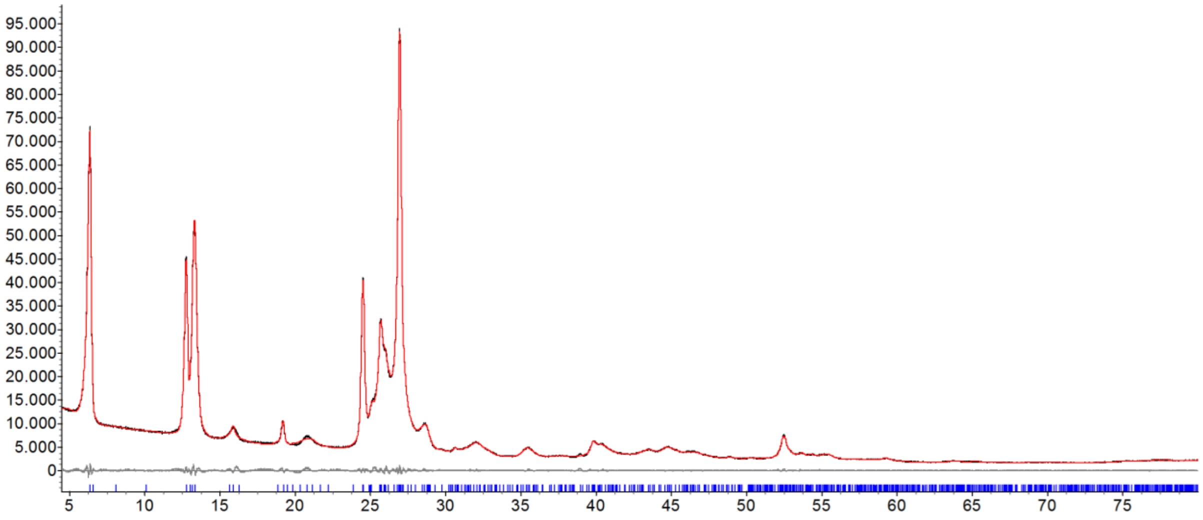

Model D, Pawley fit

Figure S2. Pawley plots. Observed intensities are denoted as black line, calculated intensities as a red line, difference curve below in grey. Vertical ticks represent the reflection positions. R_{wp} values of the Pawley fits:

A: 2.179 (Z = 4)

B: 2.170 (Z = 2)

C: 2.313 (Z = 1)

D: 2.036 (Z = 4)

Why the simulated powder patterns so similar?

(a)

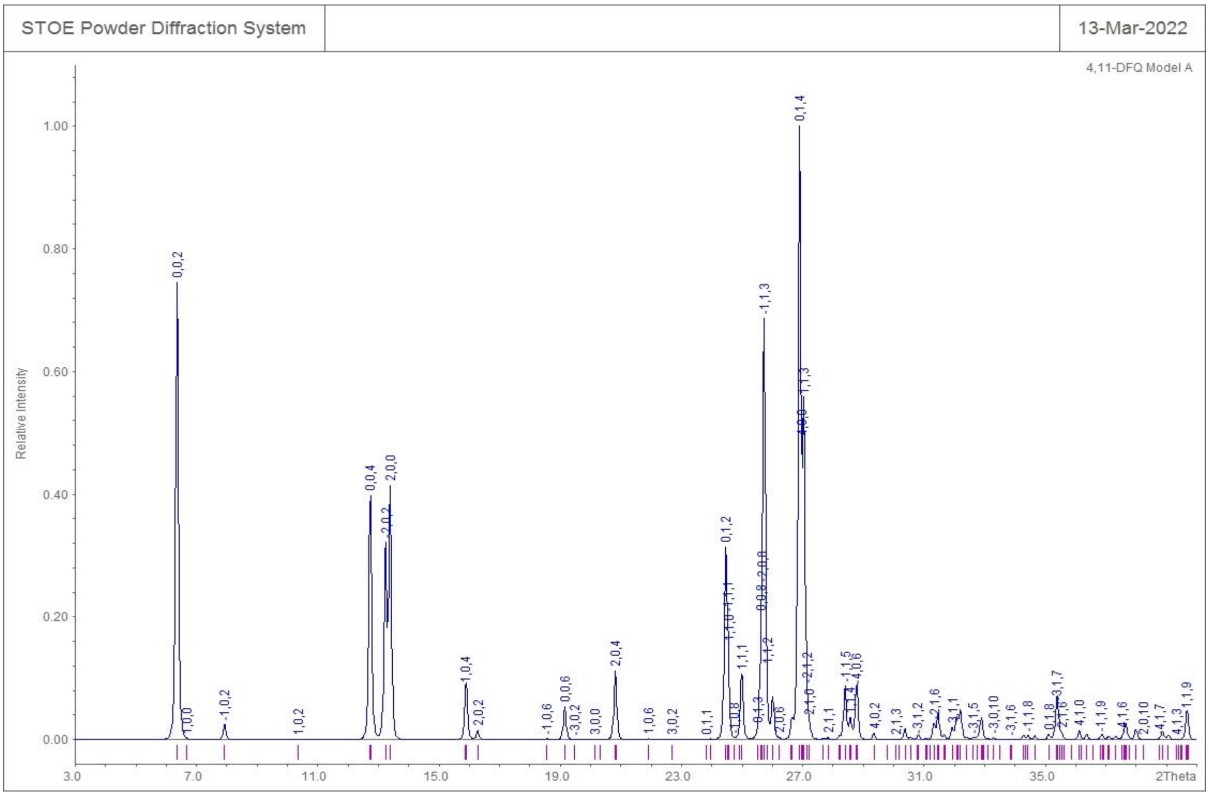

(b)

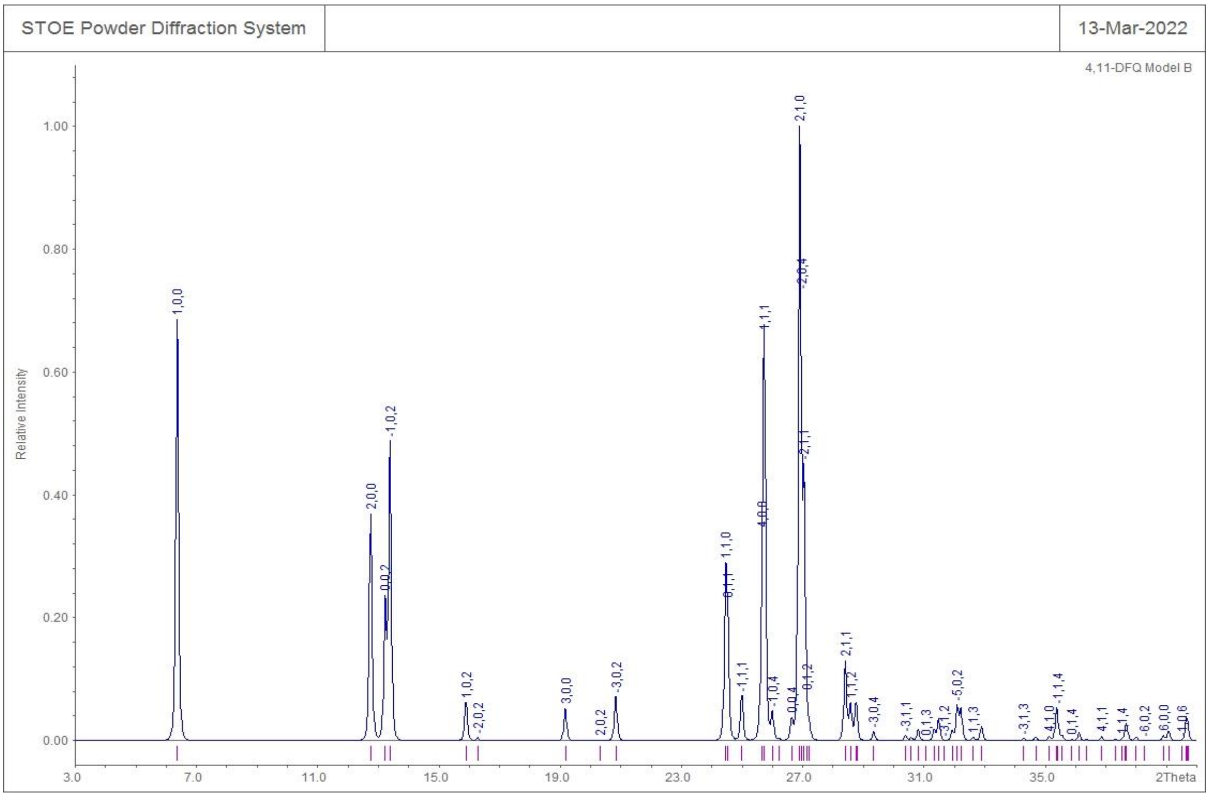
STOE Powder Diffraction System
13-Mar-2022
4,11-DFQ Model B
Relative Intensity
1.00
0.80
0.60
0.40
0.20
0.00
3.0
7.0
11.0
15.0
19.0
23.0
27.0
31.0
35.0
2Theta
1,0,0
2,0,0
-1,0,2
0,0,2
1,0,2
-2,0,2
3,0,0
2,0,2
-3,0,2
1,1,0
0,1,1
-1,1,1
1,1,1
4,0,0
-1,0,4
0,0,4
2,1,0
-2,0,4
-2,1,1
0,1,2
2,1,1
1,1,2
-3,0,4
-5,0,2

(c)

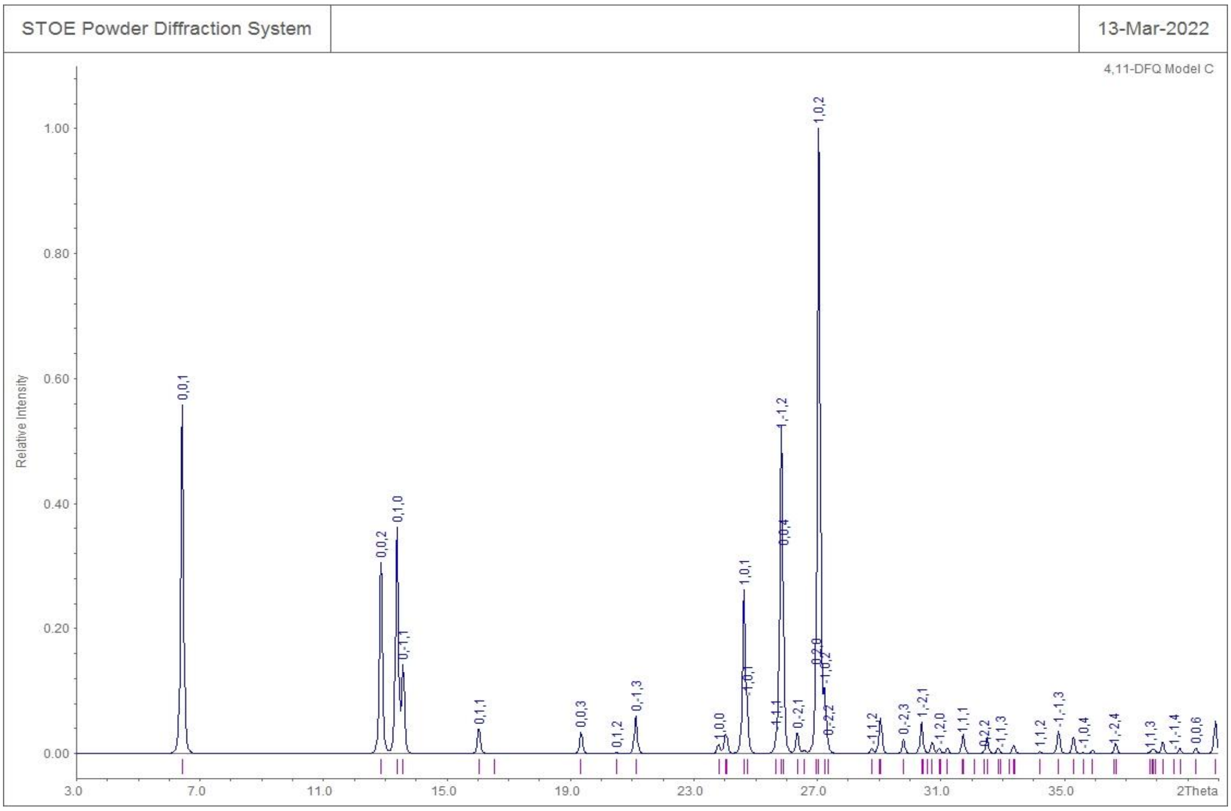
STOE Powder Diffraction System
13-Mar-2022
4,11-DFQ Model C
Relative Intensity
1.00
0.80
0.60
0.40
0.20
0.00
3.0
7.0
11.0
15.0
19.0
23.0
27.0
31.0
35.0
2Theta
0,0,1
0,0,2
0,1,0
0,-1,1
0,1,1
0,0,3
0,1,2
0,-1,3
1,0,0
1,0,1
-1,0,1
1,-1,2
0,0,4
1,-1,1
0,-2,1
1,0,2
0,2,0
-1,0,2
0,-2,2
0,-2,3
1,-2,1
1,-1,3
1,1,1
-1,0,4
1,-2,4

(d)

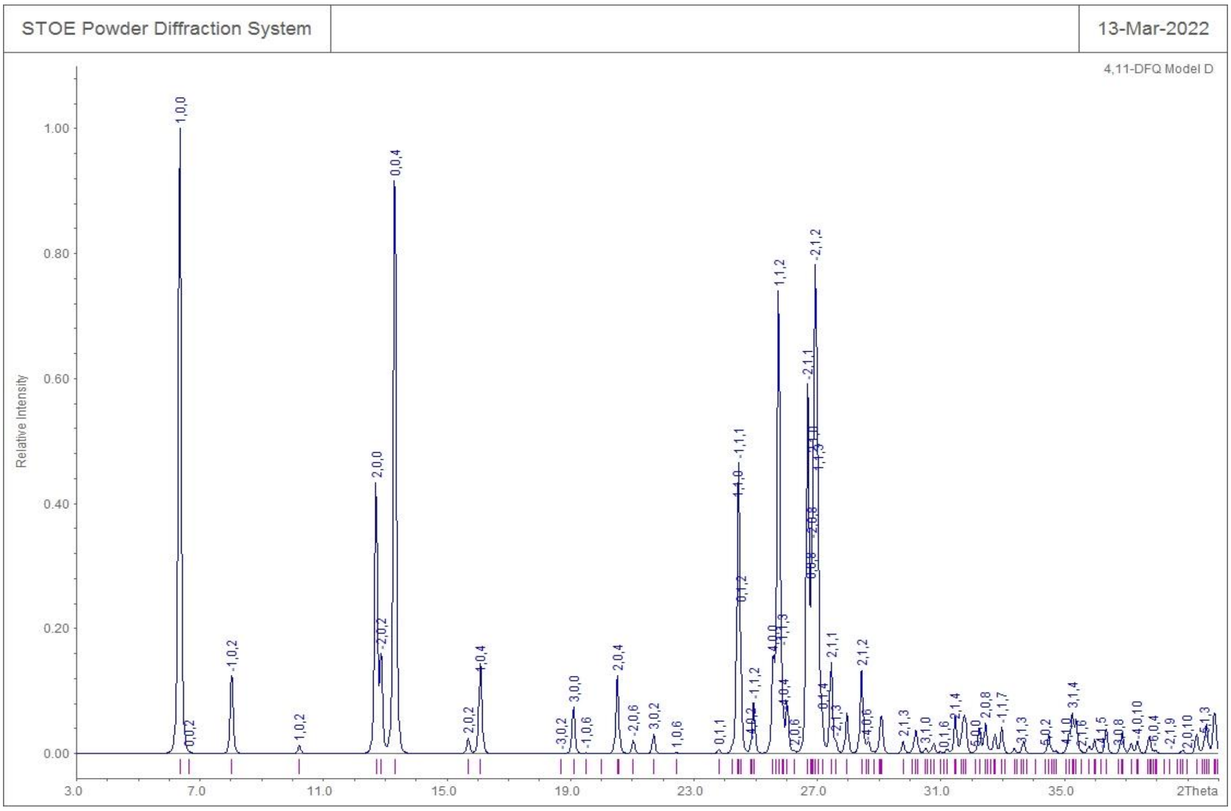

Figure S3. Simulated powder patterns of (a) Model A, (b) Model B, (c) Model C, (d) Model D. The powder patterns were simulated from the final Rietveld-refined structures without applying a correction for preferred orientation. The Miller indices correspond to the unit cells of the respective model. It can be seen that the patterns are not dominant-zone patterns.

<u>Results of the structure refinement by fit to the PDF</u>

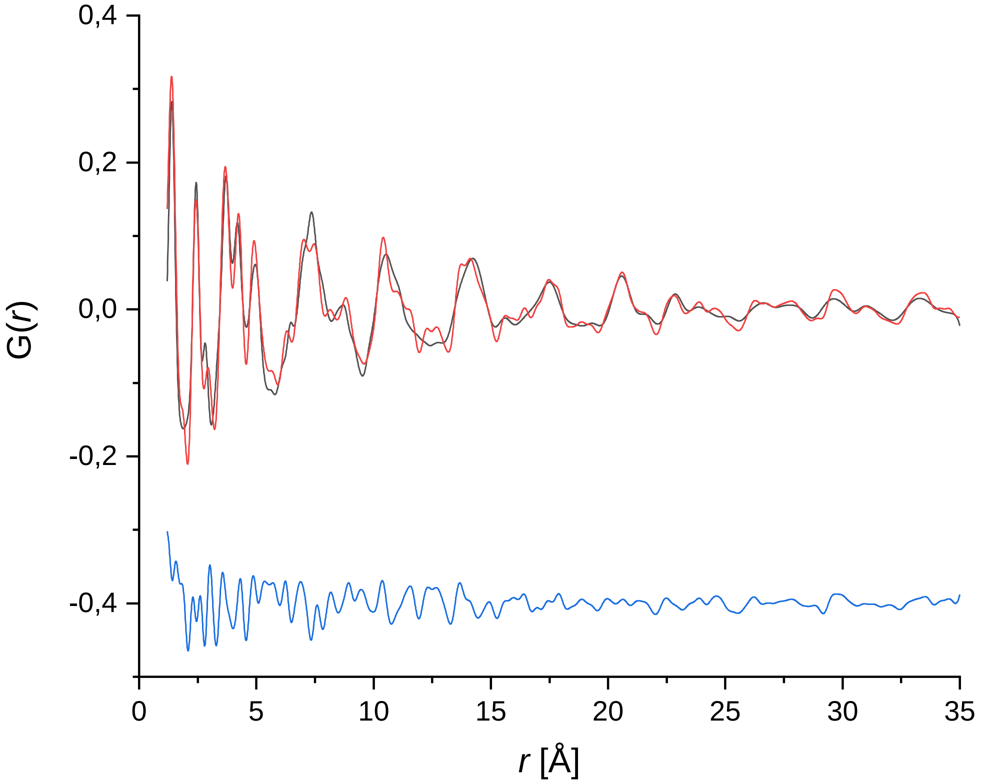

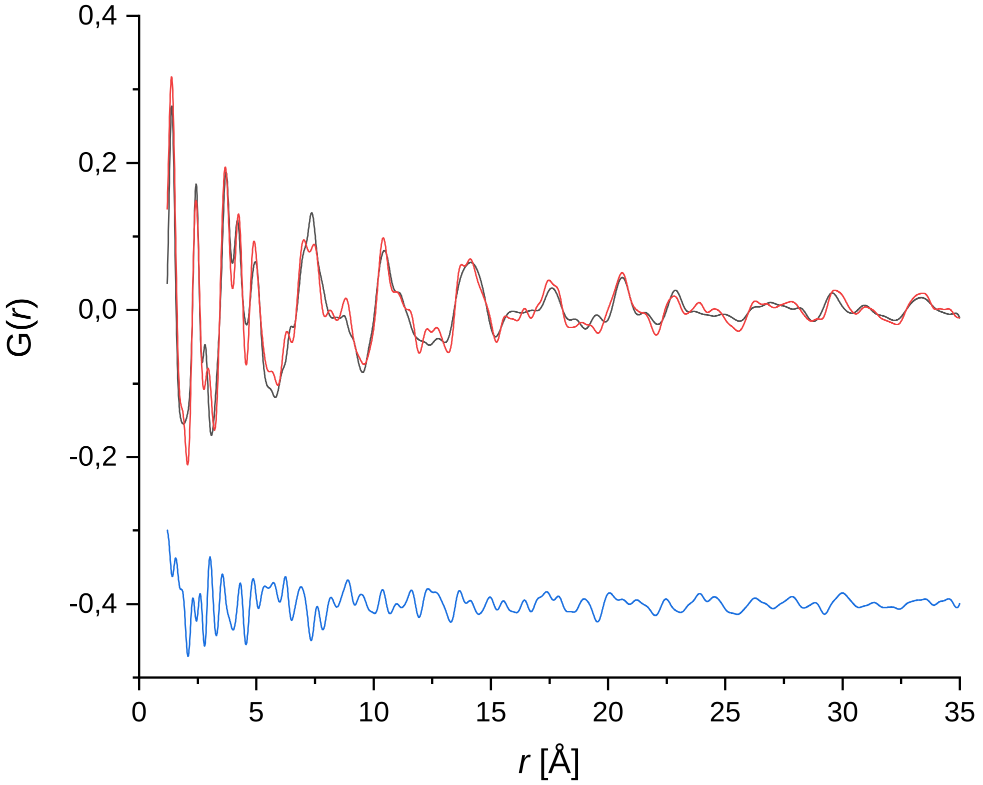

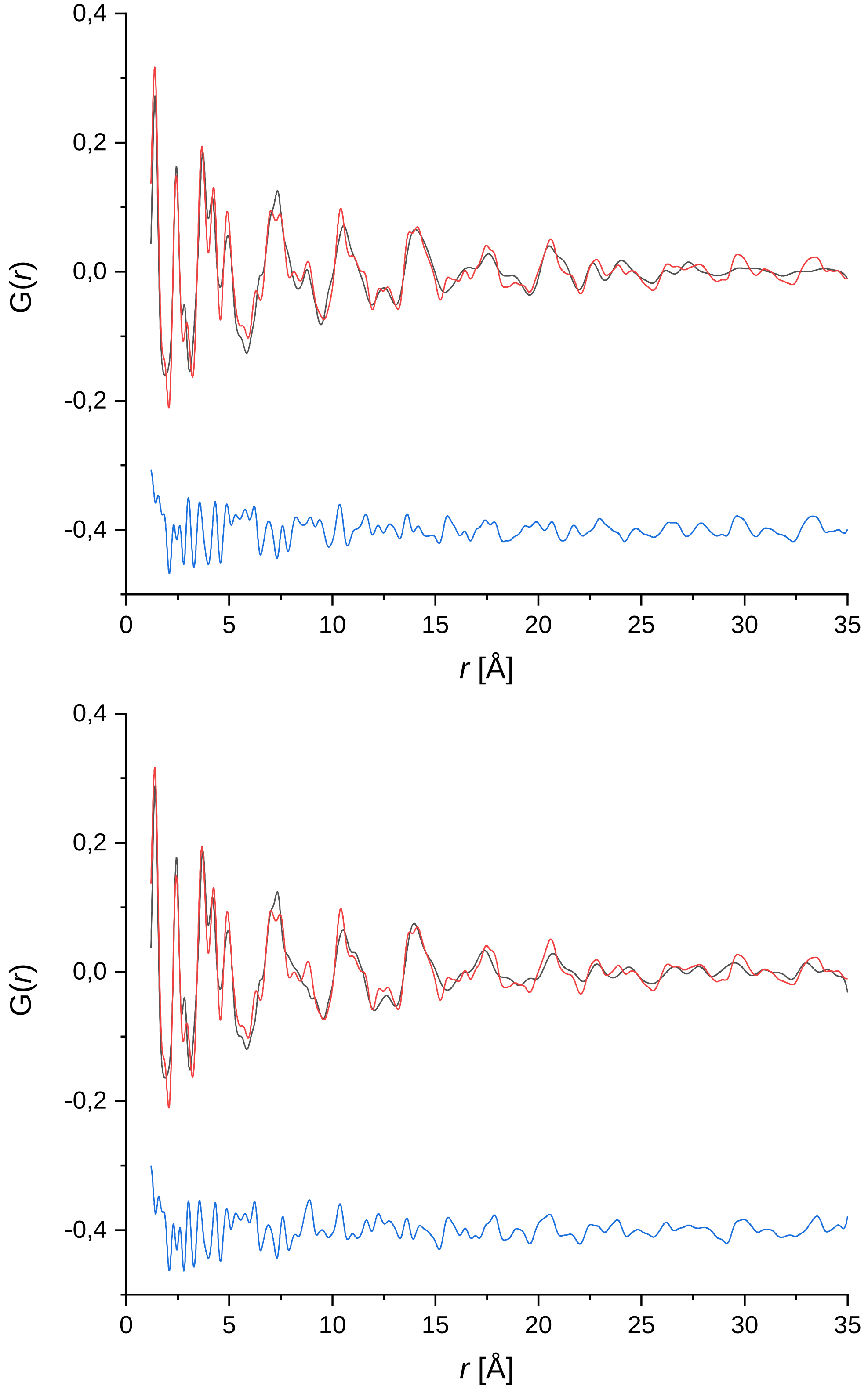

Figure S4. Crystal structure refinement of models A–D by fit to the PDF using the rigid-body approach with the 173 K data. From top to bottom: Models A, B, C. D. Observed PDF as red line, calculated PDF as black line and difference curve below in blue.

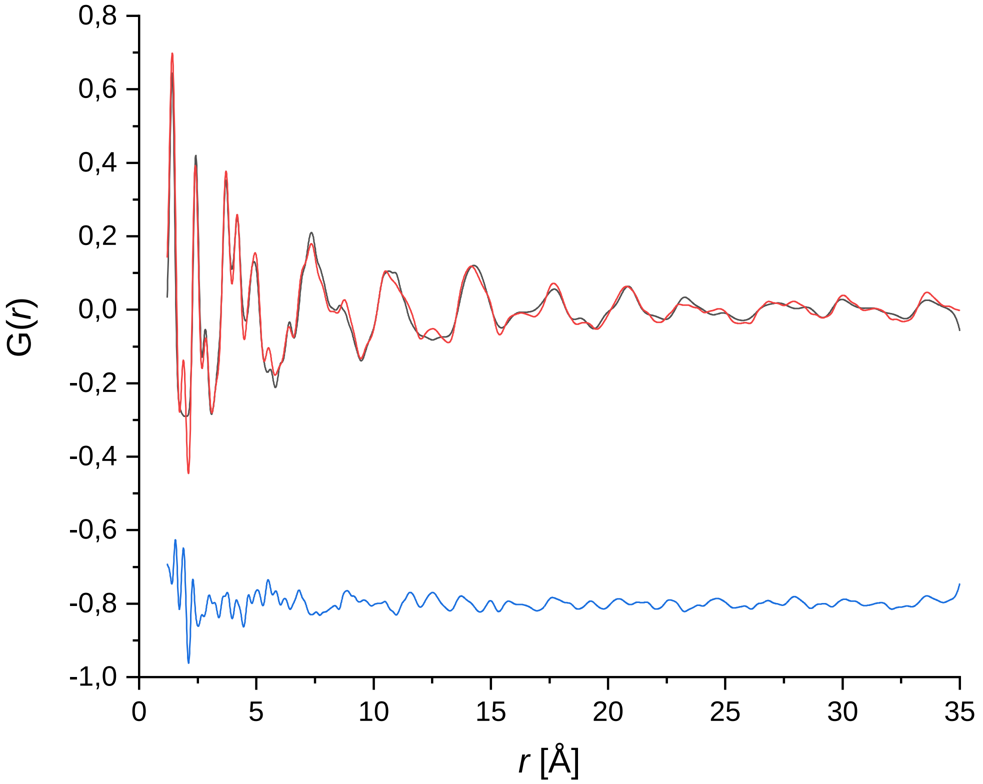
0,8
0,6
0,4
0,2
0,0
-0,2
-0,4
-0,6
-0,8
-1,0
G(r)
0
5
10
15
20
25
30
35
r [Å]

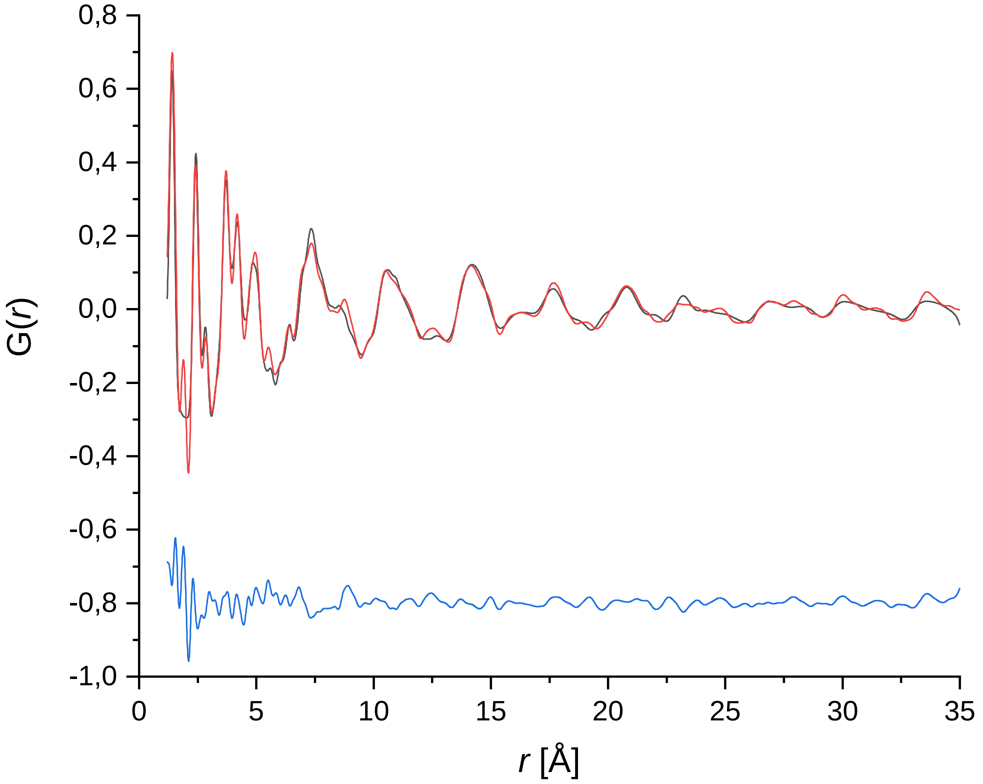
0,8
0,6
0,4
0,2
0,0
-0,2
-0,4
-0,6
-0,8
-1,0
G(r)
0
5
10
15
20
25
30
35
r [Å]

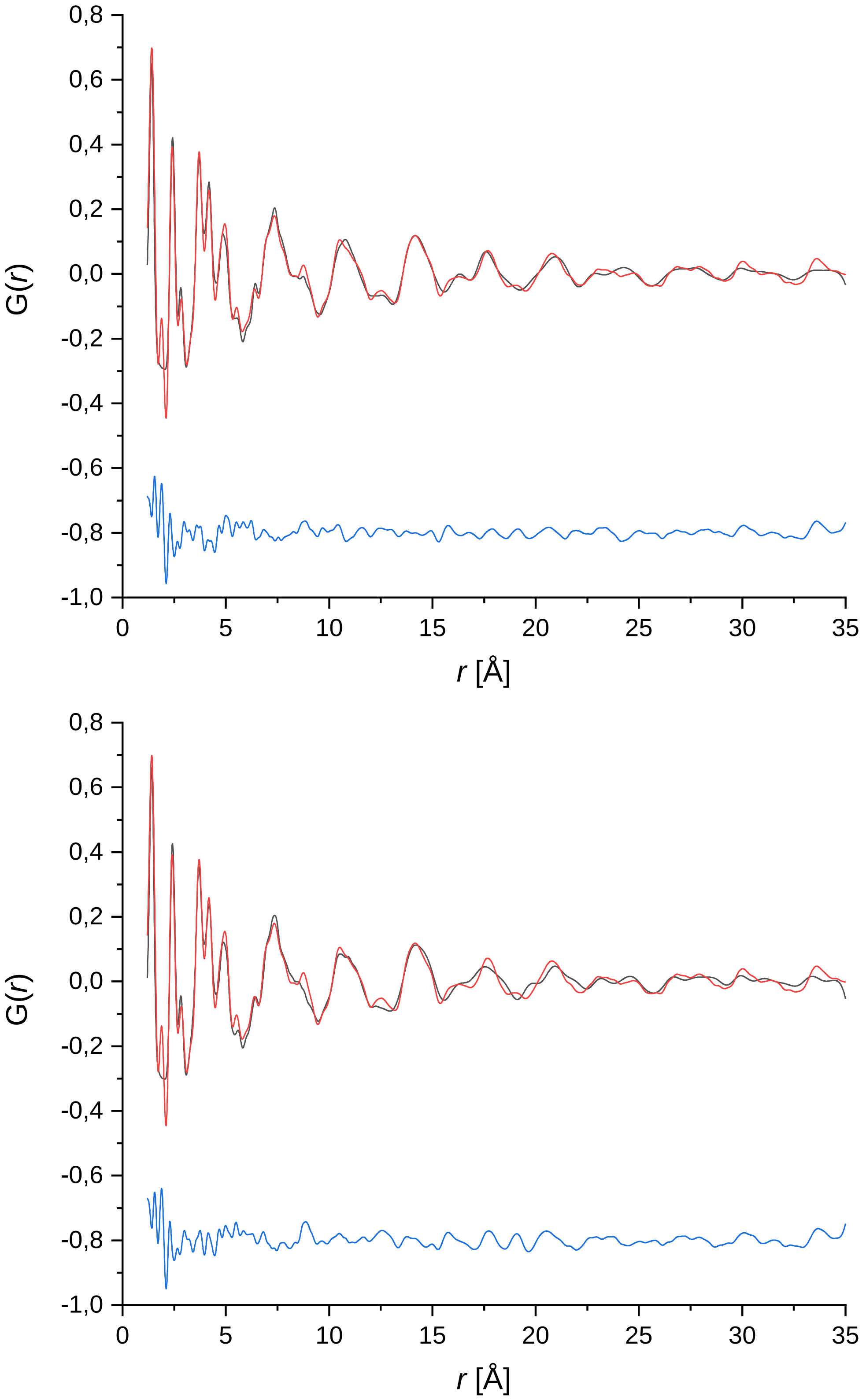

Figure S5. Crystal structure refinement of models A–D by fit to the PDF using restrain with the room-temperature data. From top to bottom: Models A, B, C. D. Observed PDF as red line, calculated PDF as black line and difference curve below in blue.

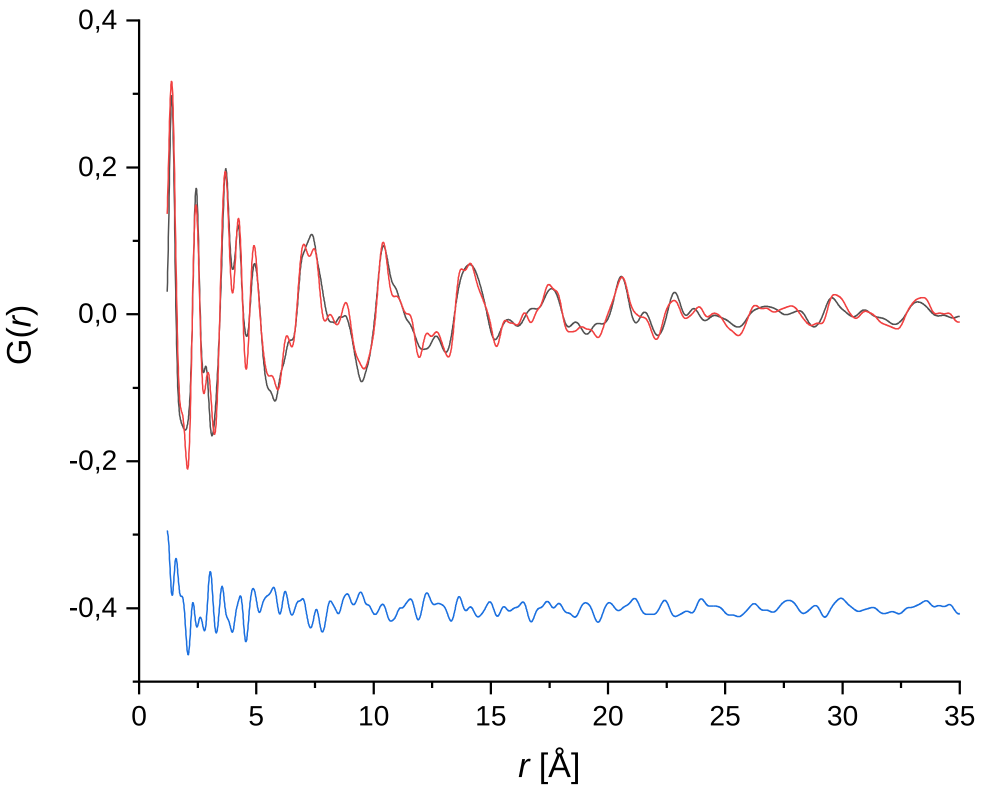
0,4
0,2
0,0
-0,2
-0,4
G(r)
0
5
10
15
20
25
30
35
r [Å]

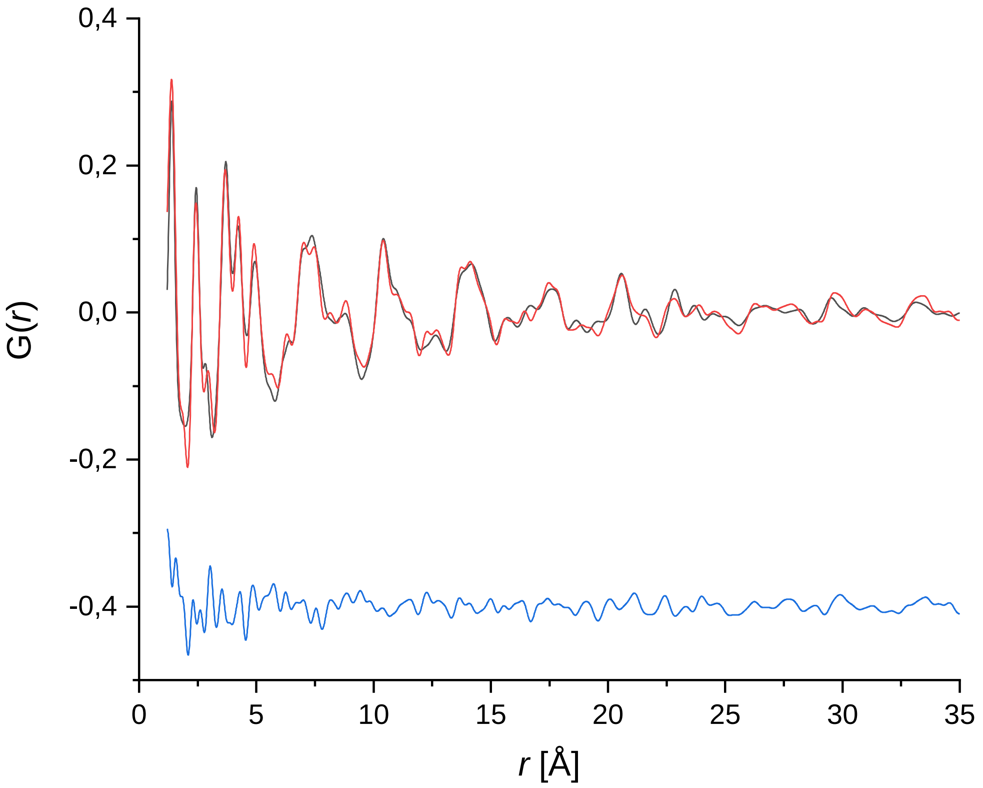
0,4
0,2
0,0
-0,2
-0,4
G(r)
0
5
10
15
20
25
30
35
r [Å]

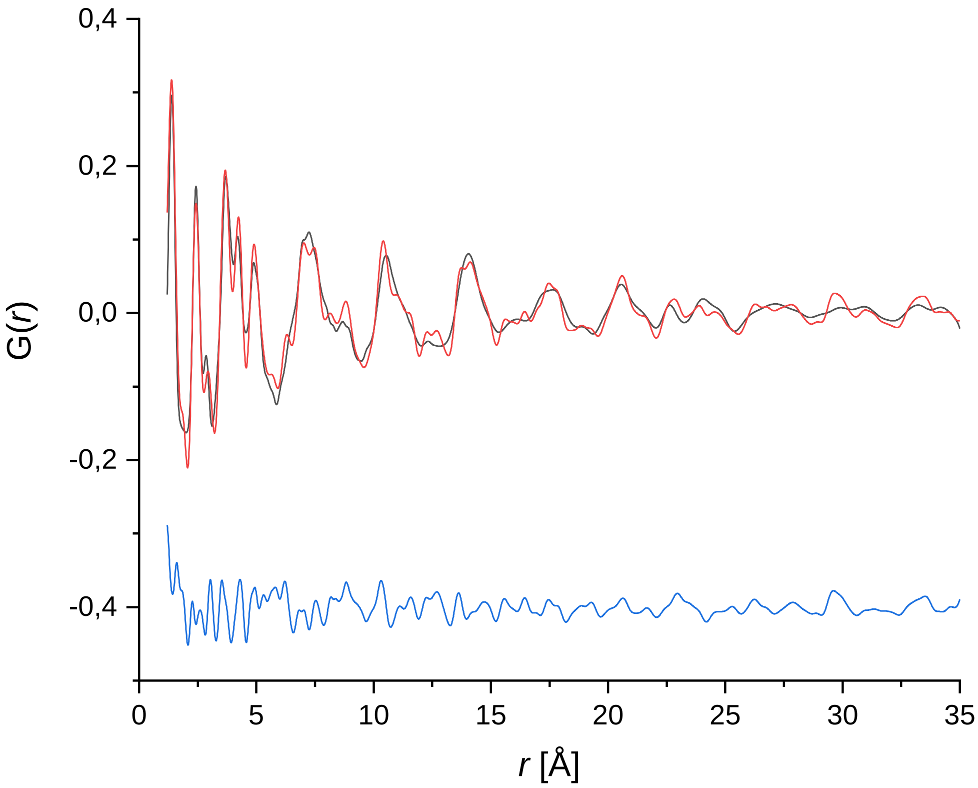

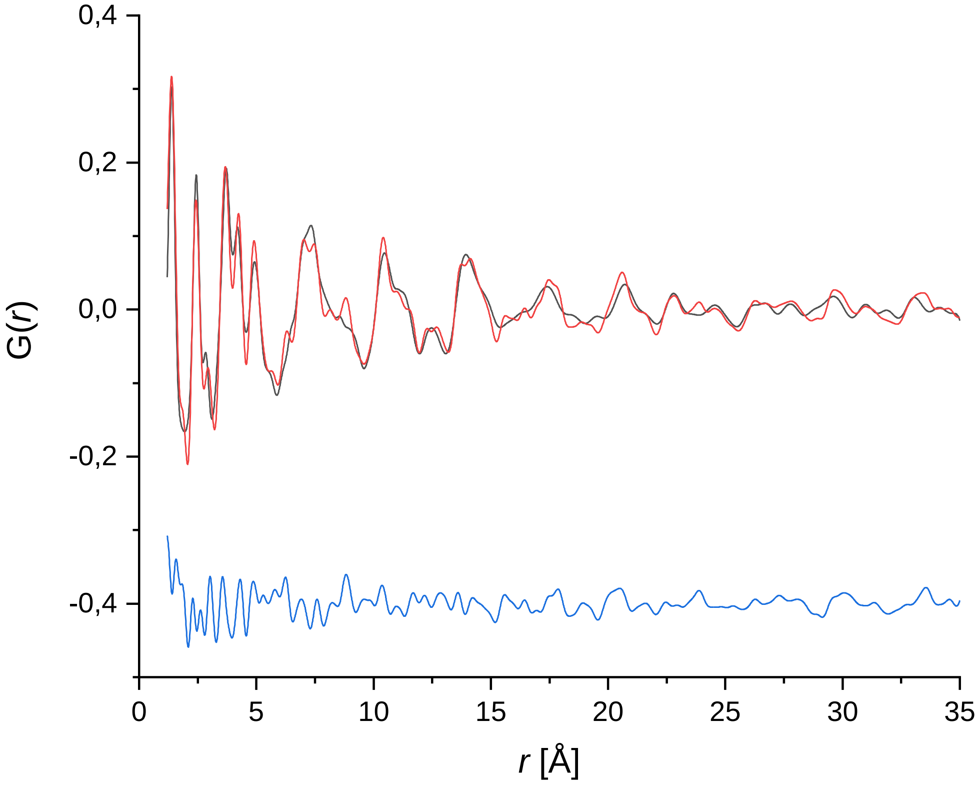

Figure S6. Crystal structure refinement of models A–D by fit to the PDF using restrains with the 173 K data. From top to bottom: Models A, B, C. D. Observed PDF as red line, calculated PDF as black line and difference curve below in blue.

Results of the lattice-energy minimisations with Quantum Espresso

(a) (b)

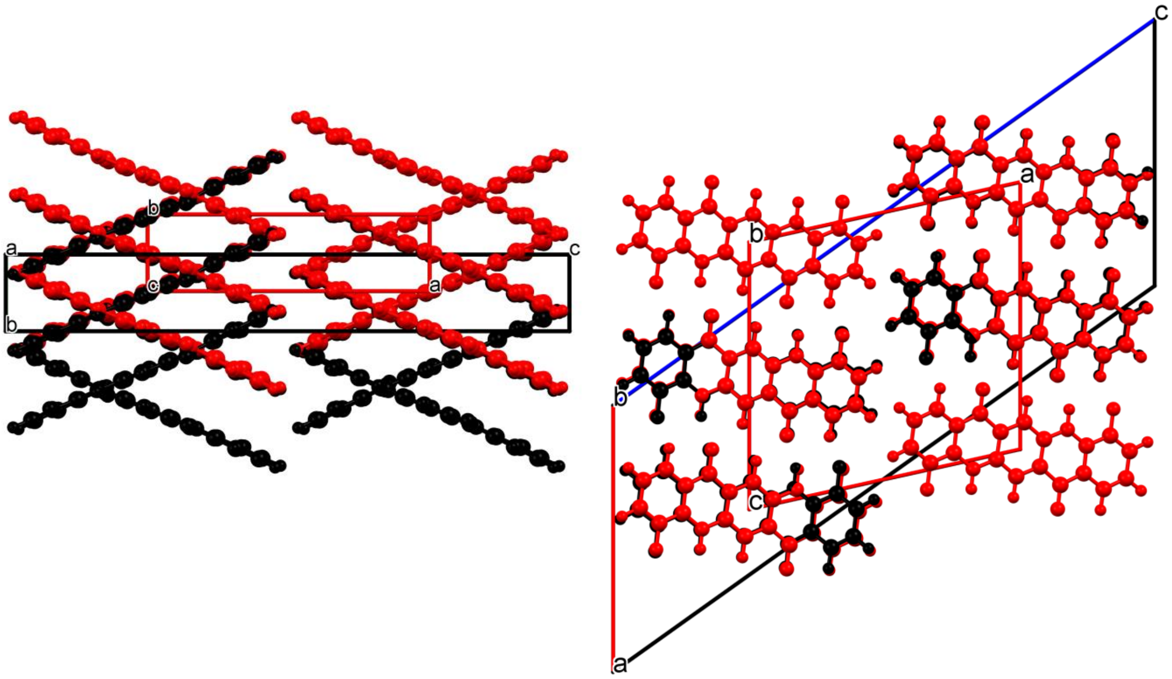

Figure S7. Lattice-energy optimizations with Quantum Espresso, with fixed lattice parameters. superpositions of models A and B. Model A shown in black, model B in Red. (a) View along the hydrogen bonds. (b) View direction [010].

Solid-state NMR investigations

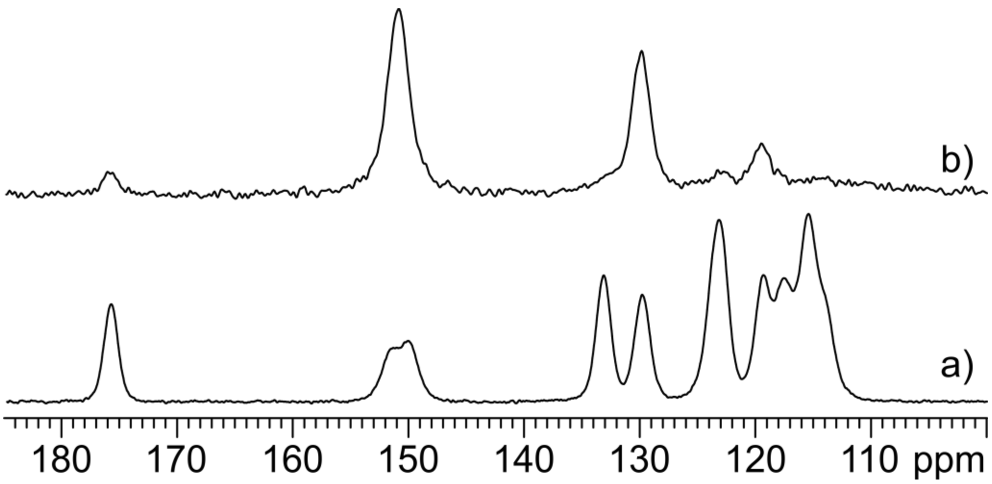

Figure S8.

a) ^{1}H-^{13}C (150.9 MHz) CPMAS SSNMR spectrum acquired at a spinning speed of 20 kHz.

b) ^{19}F-^{13}C (100.6 MHz) CPMAS SSNMR spectrum acquired at a spinning speed of 12 kHz.

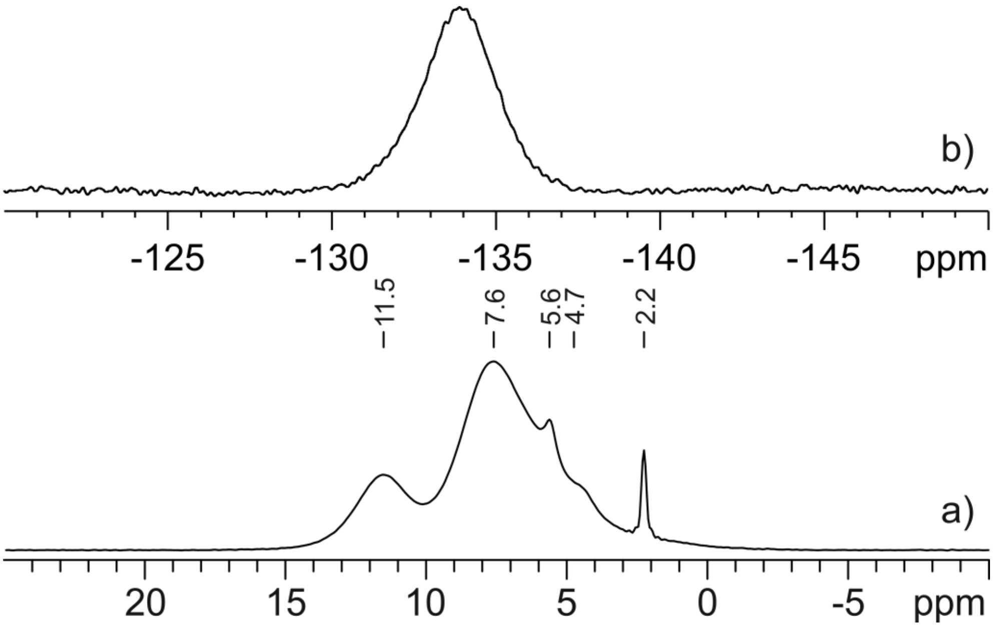

Figure S9.

a) ^{1}H (400.2 MHz) MAS SSNMR spectrum of DFQ acquired at a spinning speed of 32 kHz. The peaks at about 2.2, 4.7 and 5.6 ppm are caused by impurities.

b) ^{19}F (376.6 MHz) MAS SSNMR spectrum of DFQ acquired at a spinning speed of 32 kHz

Table S1: Experimental and computed ^{1}H and ^{13}C SSNMR chemical shifts with assignments of DFQ and ^{1}H and ^{13}C RMS values. The ^{13}C chemical shifts were experimentally observed in the ^{19}F-^{13}C{^{19}F} CPMAS spectrum (see Figure S8b). The inset shows the atomic numbering.

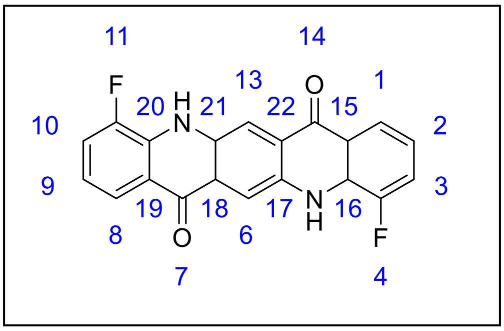

			**Model A**		**Model B**		**Model C**		**Model D**	
**^{1}H RMS**			0.82		0.79		1.16		0.85	
**^{13}C RMS**			2.28		2.29		2.37		2.39	
**Experimental**			**Model A**		**Model B**		**Model C**		**Model D**	
**#**	**^{13}C**	**^{1}H**	**^{13}C**	**^{1}H**	**^{13}C**	**^{1}H**	**^{13}C**	**^{1}H**	**^{13}C**	**^{1}H**
10	115.4	7.6	113.32	6.85	113.54	7.00	114.16	7.79	111.93	6.70
3			113.83	6.97					113.52	7.19
13			115.24	8.23	115.78	8.29	116.92	9.18	116.09	8.41
6			116.04	8.26					116.18	9.00
2	117.5		118.23	6.33	118.31	6.39	117.34	6.74	117.69	6.54
9			118.38	6.41					119.42	6.61
19	119.3	-	119.35	-	119.43	-	120.89	-	119.35	-
15			119.49						120.04	
22	123.1		121.37		121.48		121.29		121.98	
18			121.50						122.28	
1		7.6	123.32	7.82	123.30	7.87	122.46	8.37	122.41	8.01
8	123.5		123.60	7.86					122.82	8.05
16	129.8	-	127.06	-	126.83	-	125.69	-	126.53	-
20			127.41						127.30	
17	133.1		131.62		131.73		131.56		131.22	
21			131.82						131.60	
4	150.8*		156.87		156.86		155.90		156.68	
11			157.07						157.18	

14	175.7		175.05		175.65		176.74		175.04	
7			175.38						176.62	
5	-	11.5	-	12.44	-	12.07	-	9.52	-	10.88
12	-			12.56						12.42

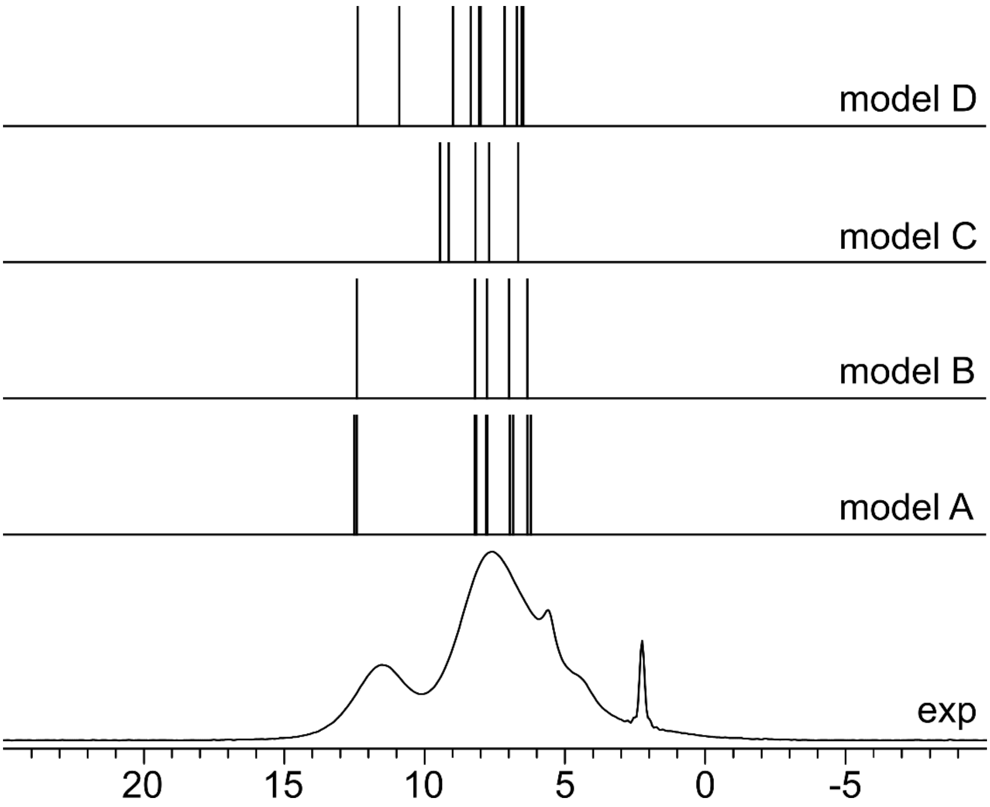

Figure S10.

Comparison between the ^{1}H (400.2 MHz) MAS experimental SSNMR spectrum of DFQ and those computed on models A, B, C and D. The peaks at about 2.2, 4.7 and 5.6 ppm are caused by impurities.

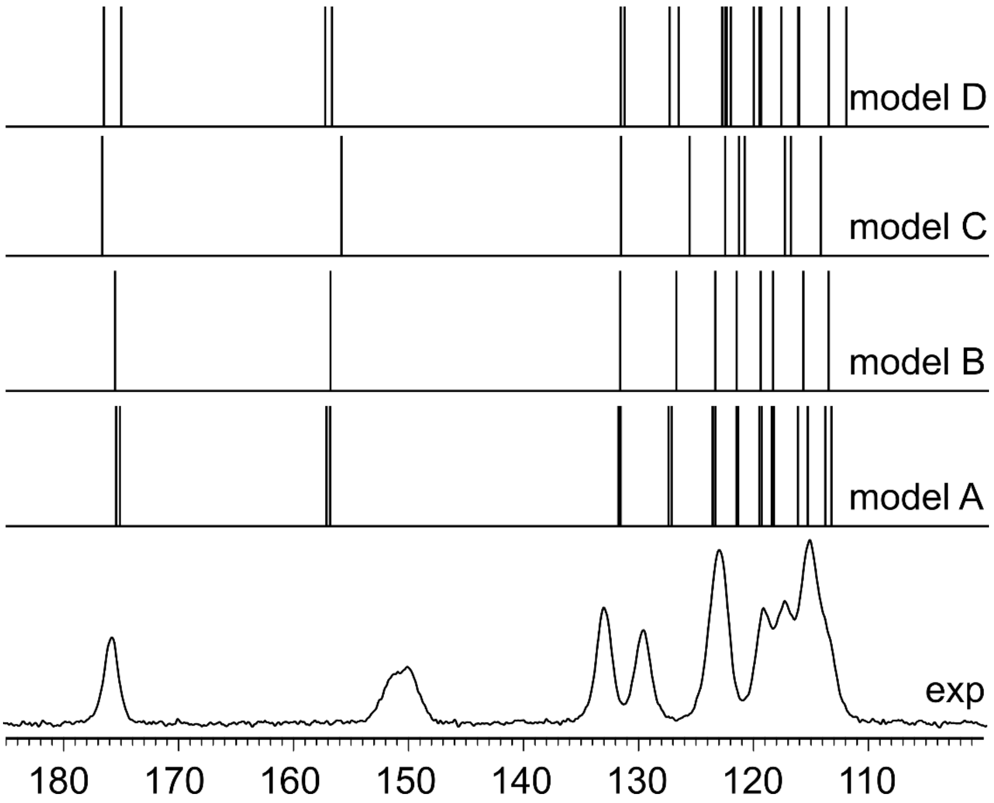

Figure S11.

Comparison between the ^{13}C (150.9 MHz) CPMAS experimental SSNMR spectrum of DFQ and those computed on models A, B, C and D.
